# Novel Dual Acetyl- and Butyrylcholinesterase Inhibitors Based on the Pyridyl–Pyridazine Moiety for the Potential Treatment of Alzheimer’s Disease

**DOI:** 10.3390/ph17101407

**Published:** 2024-10-21

**Authors:** Mohamed Elsawalhy, Adel A-H Abdel-Rahman, Ebtesam A. Basiony, Salma A. Ellithy, Allam A. Hassan, Eman S. Abou-Amra, Abdelhamid Ismail, Abdulrahman A. Almehizia, Mohamed A. Al-Omar, Ahmed M. Naglah, Nasser A. Hassan

**Affiliations:** 1 Department of Chemistry, Faculty of Science, Menofia University, Shbien El-Kom 32511, Egypt; mohamed_elsawalhy22@yahoo.com (M.E.); adelnassar63@yahoo.com (A.A.-H.A.-R.); ebtesambasiony@gmail.com (E.A.B.); salma_yousf_26@yahoo.com (S.A.E.); abdelhameed.ismail@science.menofia.edu.eg (A.I.); 2 Department of Chemistry, Faculty of Science, Suez University, Suez 43221, Egypt; allam.hassan@sci.suezuni.edu.eg; 3 Department of Chemistry, Organic Chemistry, Faculty of Science (Girls), Al-Azhar University, Cairo 11754, Egypt; emansadek.59@azhar.edu.eg; 4 Department of Pharmaceutical Chemistry, College of Pharmacy, King Saud University, Riyadh 11451, Saudi Arabia; mehizia@ksu.edu.sa (A.A.A.); malomar1@ksu.edu.sa (M.A.A.-O.); anaglah@ksu.edu.sa (A.M.N.); 5 Synthetic Unit, Department of Photochemistry, Chemical Industries Research Institute, National Research Centre, Cairo 12622, Egypt

**Keywords:** pyridazine, click chemistry, spectroscopic techniques, acetylcholinesterase, butyrylcholinesterase, Alzheimer’s disease

## Abstract

**Background**: Alzheimer’s disease (AD) is characterized by cholinergic dysfunction, making the inhibition of acetylcholinesterase (AChE) and butyrylcholinesterase (BuChE) critical for improving cholinergic neurotransmission. However, the development of effective dual inhibitors remains challenging. **Objective**: This study aims to synthesize and evaluate novel pyridazine-containing compounds as potential dual inhibitors of AChE and BuChE for AD treatment. **Methods**: Ten novel pyridazine-containing compounds were synthesized and characterized using IR, ^1^H NMR, and ^13^C NMR. The inhibitory activities against AChE and BuChE were assessed in vitro, and pharmacokinetic properties were explored through in silico ADME studies. Molecular dynamics simulations were performed for the most active compound. **Results**: Compound **5** was the most potent inhibitor, with IC_50_ values of 0.26 µM for AChE and 0.19 µM for BuChE, outperforming rivastigmine and tacrine, and showing competitive results with donepezil. Docking studies revealed a binding affinity of −10.21 kcal/mol to AChE and −13.84 kcal/mol to BuChE, with stable interactions confirmed by molecular dynamics simulations. In silico ADME studies identified favorable pharmacokinetic properties for compounds **5**, **8**, and **9**, with Compound 5 showing the best activity. **Conclusions**: Compound **5** demonstrates strong potential as a dual cholinesterase inhibitor for Alzheimer’s disease, supported by both in vitro and in silico analyses. These findings provide a basis for further optimization and development of these novel inhibitors.

## 1. Introduction

Alzheimer’s disease (AD) is a complex and progressive neurodegenerative disorder characterized by neurofibrillary tangles [1], Aβ1-42 deposition [2], oxidative stress [3], neuroinflammation [4], and metal ion dysregulation, all contributing to neuronal loss [5]. This disease affects a significant portion of the geriatric and adult populations worldwide [6,7], leading to various cognitive, behavioral, mood, and psychological impairments that exhibit variability in age of onset and clinical decline rate [8,9,10,11]. The precise etiology of AD remains elusive, with factors such as inflammation, reduced acetylcholine (ACh) concentration, β-amyloid plaque formation, τ-protein aggregation, and oxidative stress implicated in its progression [12]. A critical aspect of memory loss in AD is the decline in cholinergic activity due to ACh degradation by acetylcholinesterase (AChE).

Current treatment strategies primarily rely on AChE inhibitors to increase ACh levels and slow disease progression [12,13,14]. However, traditional single-target cholinesterase inhibitors, such as donepezil, tacrine, rivastigmine, and galantamine, may not provide optimal efficacy [15], highlighting the need for multi-target approaches. While combinations like Memantine plus cholinesterase inhibitors are used in advanced AD cases, concerns about drug interactions and patient adherence remain significant challenges [16]. In response, there is growing interest in developing dual inhibitors that target both AChE and butyrylcholinesterase (BuChE), as this strategy may enhance cholinergic transmission in AD patients [17,18]. These dual inhibitors are designed to simultaneously inhibit both enzymes, potentially offering a more effective therapeutic solution by improving neurotransmission and reducing ACh breakdown. This innovative approach marks a significant evolution in AD drug discovery, focusing on agents that target multiple cholinesterases to provide synergistic benefits in treatment.

**Figure 1:**
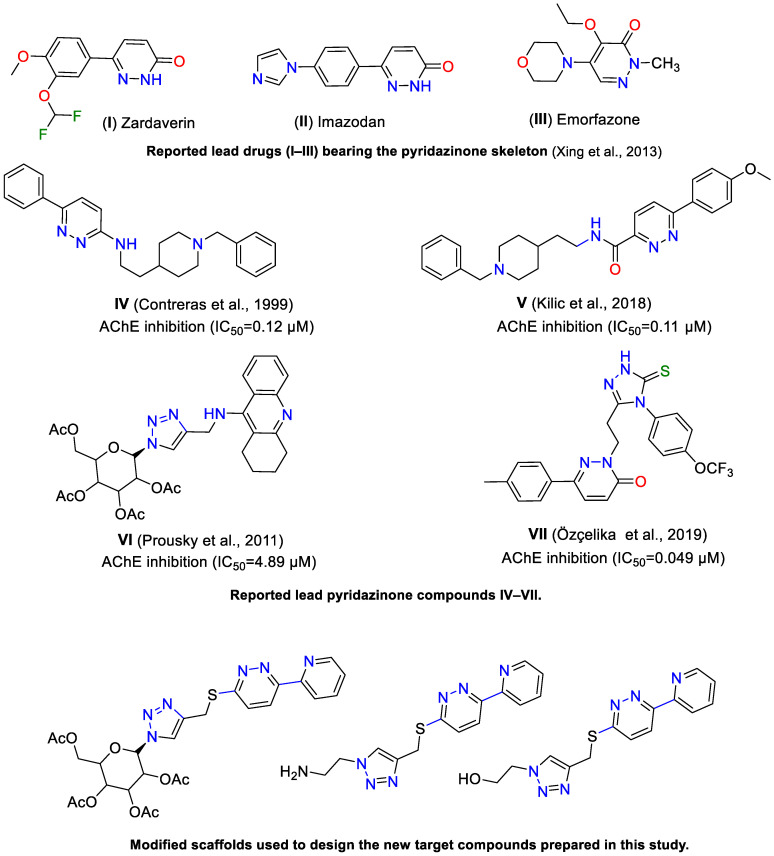
Design strategy for the new pyridyl–pyridazine as dual inhibitors of AChE and BuChE, based on the studies by Xing et al. (2013) [37], Contreras et al. (1999) [30], Kilic et al. (2018) [34], Prousky et al. (2011) [38], and Özçelika et al. (2019) [36].

In this context, pyridazine derivatives are emerging as promising candidates due to their unique chemical properties. The pyridazine ring distinguishes itself from its analogs, pyrimidine and pyrazine, by having two adjacent nitrogen atoms, which influence its electronic distribution, reactivity, and molecular interactions. This structure results in increased reactivity and enhanced biological activity. Electronic density maps indicate that pyridazine has a superior capacity for hydrogen bonding and beneficial pharmacokinetic properties [19]. Unlike pyridazinones, which contain a carbonyl group, the purely heterocyclic structure of pyridazine gives it distinct advantages. Pyridazines have demonstrated a broad spectrum of biological activities, establishing themselves as versatile candidates for various therapeutic applications, including antidepressants, antihypertensives, anticonvulsants, antibacterials, diuretics, anti-HIV agents, anticancer agents, anti-inflammatory drugs, analgesics, cardiovascular agents, and neuroprotective agents [20,21,22,23,24,25,26,27,28,29,30]. Notably, certain pyridazine derivatives, such as zardaverine, imazodane (cardiotonic PDE III inhibitors), and emorfazone (an analgesic agent), have been clinically utilized [31,32,33] (Figure 1).

Recent studies have highlighted the pharmacological potential of pyridazines in cholinesterase inhibition, positioning them as promising candidates for treating Alzheimer’s disease (AD) and other neurodegenerative disorders. For instance, Dogruer et al. investigated carboxamide and propanamide derivatives with phenylpyridazine for their inhibitory effects on cholinesterase enzymes, assessing the influence of biphenyl substitutions [34]. Similarly, Zhou et al. explored coumarin-like compounds with phenylpiperazine substitutions for their potential to inhibit acetylcholinesterase, suggesting their utility in AD treatment [35]. Uysal et al. synthesized 6-substituted-3-(2H)-pyridizinone-2-acetyl-2-(nonsubstituted/4-substituted benzenesulfonohydrazide) derivatives to evaluate their inhibitory effects on AChE and BuChE, focusing on dual inhibitors for AD [36]. Additionally, Xing et al. developed 2,6-disubstituted pyridazinone analogs to assess their activities as AChE and BuChE inhibitors [37]. Collectively, these studies provide compelling evidence that pyridazine derivatives can effectively target cholinesterase enzymes, aligning with our goals of exploring their potential as therapeutic agents for neurodegenerative diseases.

Pyridine derivatives have also been studied for their antioxidant and anti-inflammatory properties, emphasizing their relevance in central nervous system (CNS) diseases, as evidenced by the effectiveness of vitamin B3 (niacin) in treating dementia [38,39,40]. Natural sources of pyridine alkaloids show notable CNS activity [41], and pyridinium salts interact with AChE’s catalytic active site, making pyridine-based compounds valuable for developing cholinesterase inhibitors [42,43,44]. Furthermore, pyridyl–pyridazinethione derivatives exhibit neuroprotective effects by increasing EAAT2 protein levels in astrocytes, which regulate glutamate levels and reduce excitotoxicity associated with AD. Specifically, thiopyridazine demonstrates a dose-dependent increase in EAAT2 levels after 24 h of exposure [45].

Glycosides have shown neuroprotective and cognitive-enhancing effects in Alzheimer’s disease (AD) research [46,47,48]. The incorporation of 1,2,3-triazole in drug design allows for the rapid synthesis of hybrid molecules with improved chemical stability, pharmacokinetics, and toxicity profiles [49]. Triazole-based compounds exhibit diverse interactions with receptors and enzymes, aiding in the development of clinical drugs for various diseases, including infections, neurodegenerative conditions, and cancer [50,51,52,53,54,55,56,57,58,59]. The anticholinesterase effects of pyridazinone derivatives containing substituted 1,2,3-triazole are also well-documented [60]. Building on our research into biologically active compounds [61,62,63,64,65], this study focuses on designing and synthesizing novel pyridyl–pyridazine derivatives as dual inhibitors. By incorporating a triazole ring as a linker and adding diverse substituents, we aim to enhance cholinesterase inhibition and explore potential interactions with other key pathways in AD. This rational design approach seeks to advance AD drug discovery and develop more effective therapeutic agents.

## 2. Results and Discussion

### 2.1. Chemistry 

**Scheme 1:**
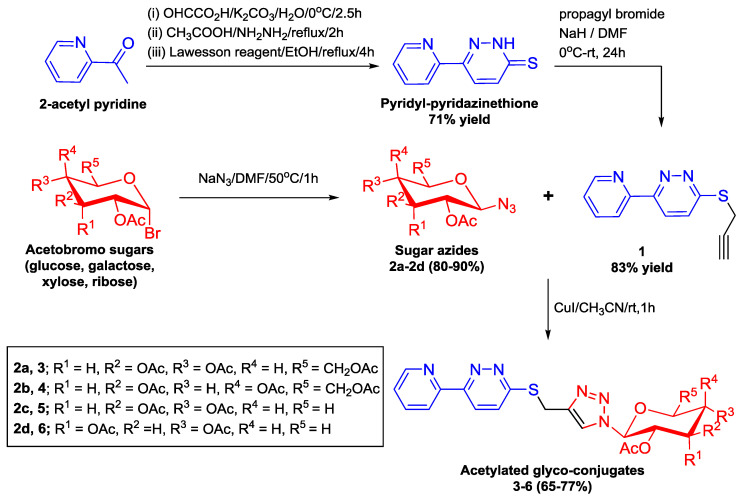
Synthesis of compounds **1**–**6**.

Click chemistry is a powerful and versatile chemical reaction used widely in various fields, including organic synthesis, materials science, and bioconjugation. Its efficiency, selectivity, and tolerance to a wide range of conditions make it a fundamental tool in modern chemical research. The most prominent example of a click reaction is the Huisgen 1,3-dipolar cycloaddition between an azide and an alkyne, catalyzed by a copper(I) species. In this context, we will discuss a synthetic protocol for preparing triazole linked pyridazine glycoconjugates through Cu(I)-catalyzed azide–alkyne cycloaddition. All the designed conjugates were successfully synthesized using Scheme 1. The azidated sugars were obtained with a high overall yield through a series of reactions involving sugar acetylation, bromination, and azidation, following the literature procedure [66,67,68]. Additionally, various alkyl azides were prepared from halo alkyl alcohols or amines in a simple one-step procedure by stirring them with a solution of sodium azide [69].

An essential intermediate in the synthesis of the title compounds is 5-(pyridazin-3-yl)pyridin-2(1H)-thione. The process of synthesizing the latter compound involves multiple steps, as illustrated in Scheme 1 [70,71]. Subsequently, the thione derivative underwent propargylation in the presence of propargyl bromide in anhydrous DMF, leading to the formation of its alkyne form **1,** which was confirmed by IR, ^1^H NMR and ^13^C NMR. The alkynyl derivative produced exhibited characteristic acetylenic absorption bands at 3314 and 2218 cm^−1^ in the IR spectrum. Additionally, the ^1^H NMR spectra showed signals corresponding to the acetylene proton of the propargyl group and the methylene protons at δ = 3.17 and 4.18 ppm, respectively. The 3-(6-(prop-2-yn-1-ylthio)pyridin-3-yl)pyridazine **1**, with a free terminal alkyne group, underwent successful click reactions with a selection of sugar azides, namely tetra-*O*-acetyl-*β*-D-gluco-, tetra-*O*-acetyl-*β*-D-galacto-, tri-*O*-acetyl-*β*-D-xylopyranosyl, and tri-*O*-acetyl-*β*-D-ribofuranosyl azides **2a**–**d**, respectively, to afford the corresponding triazole-linked pyridazine glycoconjugates **3**–**6** in good yields (65–73%) as shown in Scheme 1. The combination of elemental analyses and spectral data confirmed the structures of the prepared series **3**–**6**. As an illustration within the prepared series, compound **3** exhibited analytical data revealing a molecular formula of C_26_H_28_N_6_O_9_S (M+ 600). The infrared spectra distinctly indicated the absence of the alkyne groups present in the precursor, propargylated **1**. In the ^1^H NMR spectrum, the proton at the anomeric position in the glucose part displayed a doublet at δ 5.93 ppm, with a coupling constant of *J* = 8.8 Hz, indicating the β-configuration of the glucopyranose section connected to the 1,2,3-triazole ring. Other glucopyranose protons ranged from δ 4.01–5.52 ppm, and the four acetoxy groups appeared as singlets between 1.88 and 2.08 ppm. The triazole CH in the new 1,2,3-triazole ring was observed as a singlet at 8.00 ppm. In the ^13^C NMR spectrum, acetoxy carbonyl carbon atoms from the sugar component were detected at δ 168.9–170.5 ppm. Simultaneously, acetate methyl carbon atoms appeared at δ 20.3–20.9 ppm. The spectrum displayed six peaks from δ 62.3–84.4 ppm for the sugar chain. Peaks at δ 123.2 and 144.5 ppm represented the triazole ring, and peaks at δ 121.1, 124.8, 126.0, 127.1, 138.1, 150.0, 152.8, 156.7, and 162.8 ppm indicated the pyridine and pyridazine rings (See Supplementary Materials).

**Scheme 2:**
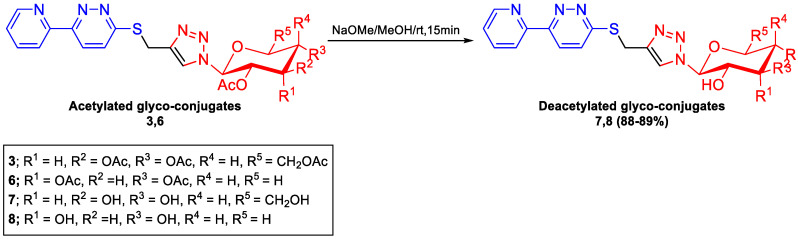
Synthesis of compounds **7**, **8**.

Deprotection of glycoconjugates **3** and **6** was achieved by treating them with sodium methoxide in dry methanol, leading to the formation of glycosides **7** and **8**, as illustrated in Scheme 2. In these glycosides, the sugar components had free hydroxyl groups, which were confirmed by their presence in the IR spectrum. The absence of acetyl methyl proton signals in the ^1^H NMR spectra of these products further confirmed the deacetylation process and validated their formation.

**Scheme 3:**
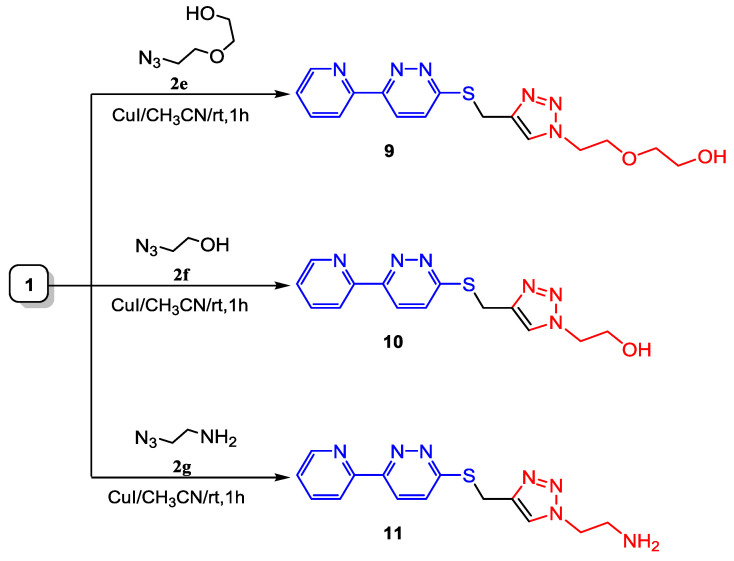
Synthesis of compounds **9**–**11**.

Similarly, in accordance with the click reaction conditions, the terminal alkyne group in compound **1** underwent a series of reactions with various acyclic azide derivatives, including 2-(2-azidoethoxy)ethan-1-ol **2e**, 2-azidoethan-1-ol **2f**, and 2-azidoethan-1-amine **2g** (see Scheme 3). This process resulted in the formation of products **9**, **10**, and **11** with yields of 72%, 69%, and 65%, respectively. The structures of these newly formed products were verified using ^1^H NMR and ^13^C NMR spectroscopy, which displayed the anticipated signals for the hydrogens and carbon atoms at their respective positions (See Section 3 and Supplementary Materials).

### 2.2. Biological Investigations

#### AChE and BuChE Inhibitory Activities

Using an in vitro Ellman’s method, all synthesized hybrids were tested for AChE and BuChE inhibition at 1 µM. Compound 5 showed the highest inhibition (AChE, 71%; BuChE, 67%), closely followed by donepezil (72%) and tacrine (67%). This concentration allows for meaningful comparisons, avoiding saturation seen at higher concentrations like 100 µM. Table S1 in the Supplementary Materials summarizes inhibitory activities at additional concentrations.

Based on the in vitro acetylcholinesterase inhibition data (IC_50_), compound **5** is the most potent, with an IC_50_ of 0.26 µM, exhibiting over ten times stronger inhibition than rivastigmine (IC_50_ = 2.76 µM) and 1.5 times more potent than tacrine (IC_50_ = 0.44 µM), though it is half as potent as donepezil (IC_50_ = 0.17 µM). Compounds **8**, **9**, and **10** also showed significant inhibition, with IC_50_ values of 0.64 µM and 1.84 µM, surpassing rivastigmine but being less effective than tacrine and donepezil. Compounds **3**, **6**, and **7** showed moderate inhibition, while the least potent compounds were **1**, **4**, and **11**, with IC_50_ values between 11.54 µM and 14.63 µM.

The in vitro butyrylcholinesterase inhibition results showed significant variation in potency among the compounds. Compounds **3** and **5** were the most potent, with compound **5** being 0.46 times and compound **3** being 0.72 times more effective than donepezil (IC_50_ = 0.41 µM) (see Table 1). Compound **9** was slightly less effective (0.93 times). Compounds **6** and **11** were the least potent, being 248.76 and 332.64 times less effective than tacrine (IC_50_ = 0.12 µM). Compared to rivastigmine (IC_50_ = 18.08 µM), compounds **3**, **5**, and **9** were 60.86 to 95.67 times more effective, while compounds **1**, **7**, **8**, and **10** were 2.4 to 5.29 times more potent. However, compounds **6** and **11** were slightly less effective than rivastigmine.

### 2.3. Docking Studies

#### Docking Study of Molecules

The described compounds **1** and **3**–**11** were docked to the active AChE site using a molecular modeling approach (PDB ID: 4EY7). In order to identify the binding modalities and interactions with the crucial amino acids, docking experiments were performed. Docking the co-crystallized ligand (donepezil) to the pocket’s active site confirmed the accuracy of the docking technique. Each ligand–protein combination in Table 2 had a negative binding energy, suggesting that the recognition process between the compounds under study and the targeted protein was thermodynamically beneficial. Binding affinities of the investigated substances ranged from −6.35 to −10.21 kcal/mol, indicating strong interactions with the targeted protein (Table 2).

**Figure 2:**
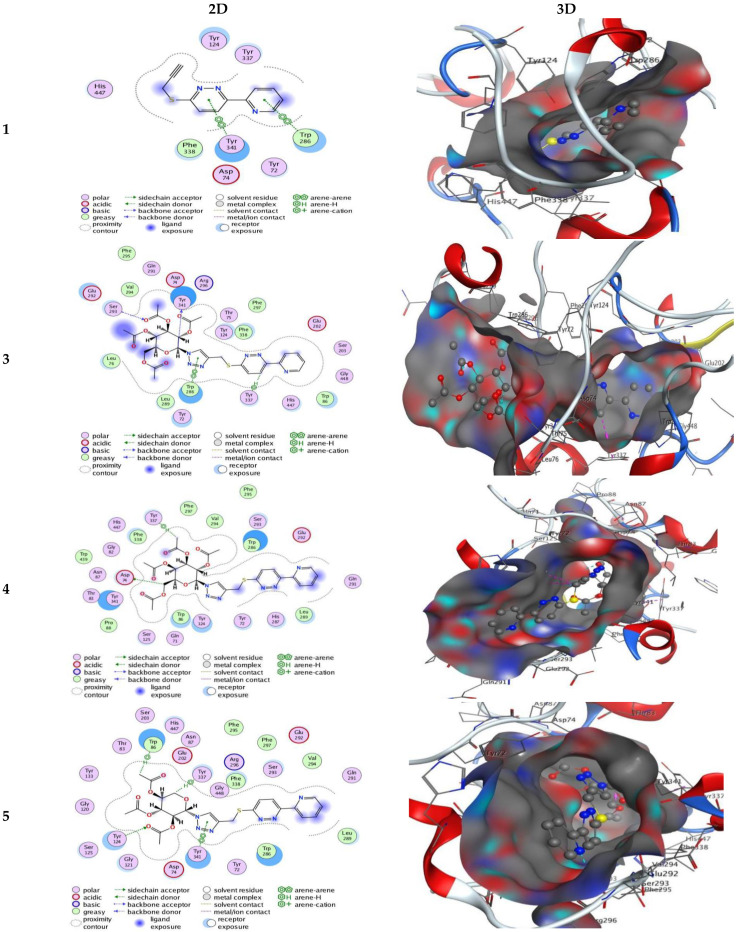

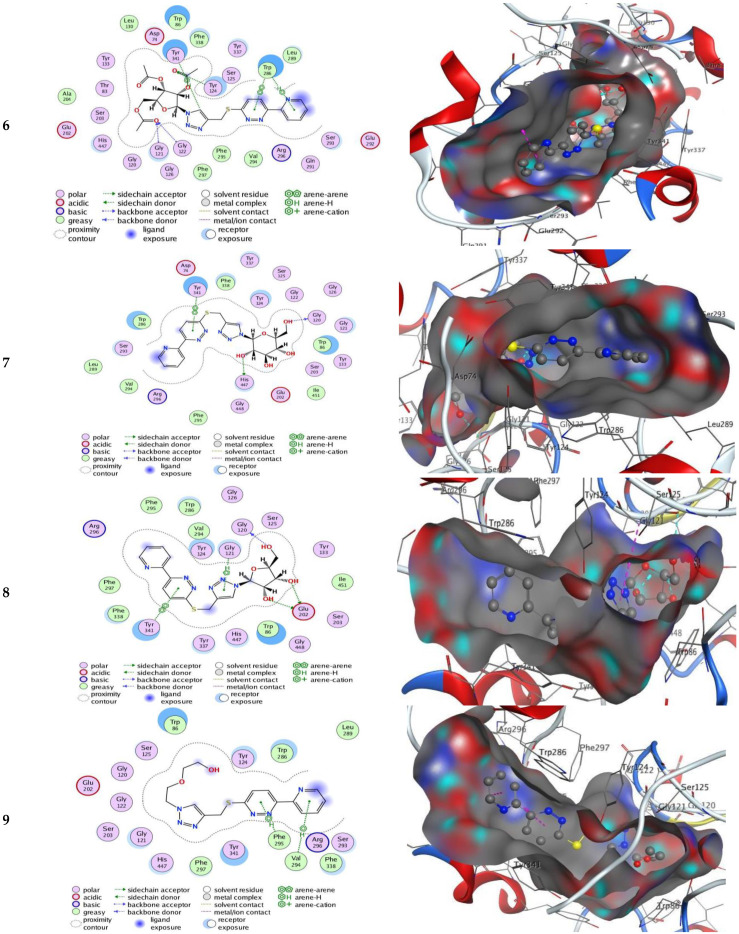

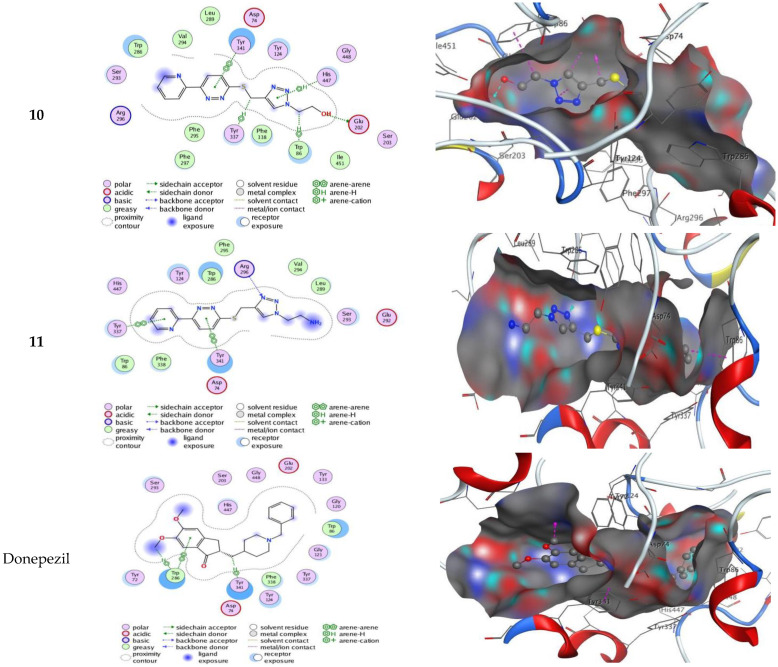
Two-dimensional and three-dimensional interactions of **1** and **3**–**11** and donepezil in the active site of AChE (PDB ID: 4EY7).

The pyridine and pyridazine rings of compound **1** were detected to have pi–pi stacking with Trp 286 and Tyr 341, respectively. The interaction of compound **3** against the target protein AChE was confirmed with Tyr 341 and Ser 293 through a pair of hydrogen bonds, with Tyr 337 through an H–pi bond, and with Trp 286 through pi–pi-stacked interaction. Compound **4** was combined with the receptor through an H-bond with Asp 74 and H–pi bond with Tyr 337 amino acid. Compound **5** showed a high binding-affinity docking score of −10.21 kcal/mol and established an H-bond with Tyr 124, pi–pi stacking between Tyr 341 amino acid with triazole ring, and two H–pi bonds with Tyr 337 and Trp 86. Five different residues on AChE were involved in the binding of **6**. It was shown to include three hydrogen bonds with Gly 121, Gly 122, and Tyr 124; an H–pi bond with Tyr 341; arene–H contact with Trp 286; and pi–pi interaction between pyridazine ring with Trp 286. The compound-**7**-predicted binding pattern identified two hydrogen bonds with amino acid residues, including Gly 120 and His 447. Further, the pyridazine ring was detected to have pi–pi stacking with Tyr 341. Compound **8** has a binding energy score of −8.26 kcal/mol, which is close to that of donepezil. It stabilizes its interaction with AChE by forming three hydrogen bonds with Gly 120 and Glu 202, along with an arene–H contact with Gly 121 and a pi–pi interaction with Tyr 341. Compound **9** has a binding-affinity score of −7.75 kcal/mol and forms two arene–H contacts with Val 294 and Phe 295 amino acids. Compound **10** interacted with Glu 202 through a hydrogen bond interaction, with Tyr 337 and Trp 86 through two H–pi bonds, with His 447 through arene–H contact, and with Tyr 341 with a pi–pi-stacked interaction. Compound **11** was coupled with the receptor protein by forming an H-bond with Arg 296 and two pi–pi interactions between Tyr 341 and Tyr 337 amino acids with the pyridazine and pyridine ring, respectively. Finally, donepezil had a binding-affinity score of −8.65 kcal/mol. It formed two H–pi bonds with Trp 286 and Tyr 341 residues, as well as a pi–pi-stacking interaction observed between the phenyl ring and Trp 286 amino acid. Figure 2 depicts the overall bonding connections as hydrogen bonds, polar, and hydrophobic contacts of the relevant amino acid residues in 4EY7 protein against the docked molecules and donepezil.

**Figure 3:**
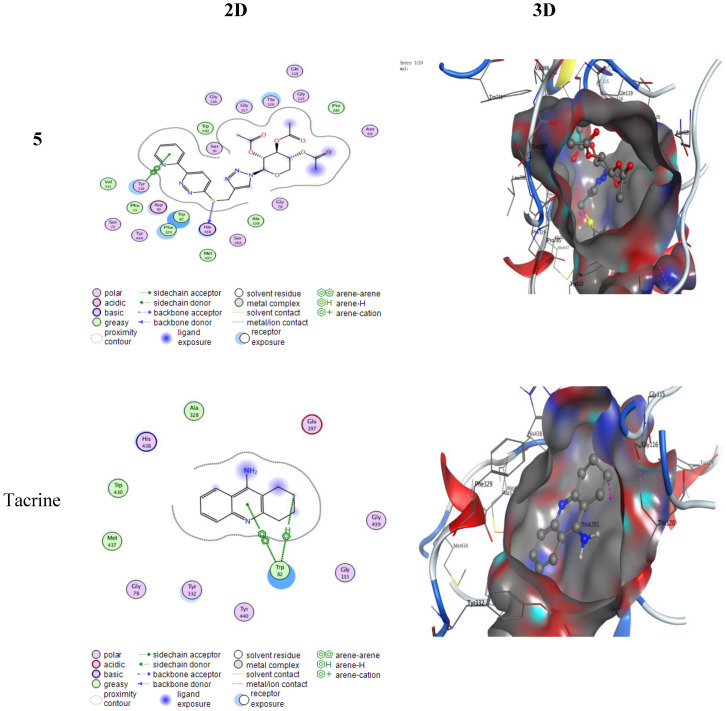
Two-dimensional and three-dimensional interactions of **5** and tacrine in the active site of BuChE (PDB ID: 4BDS).

In a similar analysis, the docking of compound **5** to the active site of butyrylcholinesterase (BuChE) was conducted to evaluate its binding interactions and affinities. Given the structural and functional differences between acetylcholinesterase (AChE) and BuChE, it is crucial to understand how these compounds interact with both enzymes to assess their potential therapeutic efficacy. Compound **5** was docked to the active BuChE site using a molecular modeling approach (PDB ID: 4BDS). To identify the binding modalities and interactions with crucial amino acids, docking experiments were performed (Figure 3). Docking the co-crystallized ligand (tacrine) to the pocket’s active site confirmed the accuracy of the docking technique. Each ligand–protein combination in Table 3 had a negative binding energy, suggesting that the recognition process between the compound under study and the targeted protein was thermodynamically beneficial. Binding energies of the investigated substances ranged from −7.45 to −13.84 kcal/mol, indicating high affinity (Table 3). Compound **5** showed a high binding-energy-docking score of −13.84 kcal/mol and established an H-bond donor interaction with His448, along with a pi–pi-stacking interaction between Tyr332 and its 6-ring. Tacrine had a binding-energy score of −7.45 kcal/mol and formed two H–pi bonds, as well as a pi–pi-stacking interaction with the Trp82 amino acid.

### 2.4. Molecular Dynamics of AChE and BuChE

**Figure 4:**
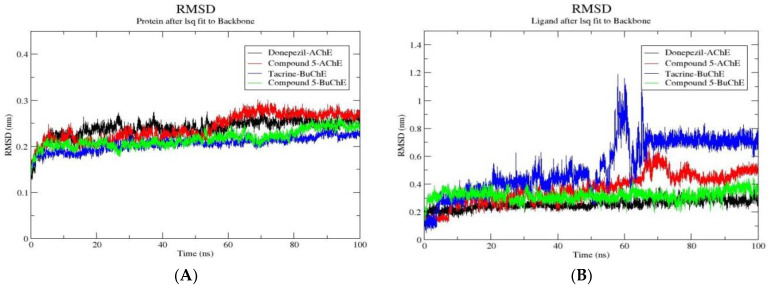
RMSDs of the target enzymes; protein backbone RMSD (**A**), AChE, BuChE, and compound **5** in complex with the 2 targets (**B**) throughout 100 ns MD simulation.

To investigate the stability of interactions between compound **5** and cholinesterase enzymes, we conducted 100-nanosecond (ns) molecular dynamics (MD) simulations using GROMACS-2023.1 [72] for both acetylcholinesterase (AChE, PDB ID: 4EY7) and butyrylcholinesterase (BuChE, PDB ID: 4BDS). The protein backbone RMSD for both enzymes (Figure 4A) indicated remarkable structural stability, with AChE fluctuations remaining under 0.05 nm and BuChE maintaining stability during binding. The ligand RMSD analysis (Figure 4B) revealed that compound **5** exhibited slightly higher fluctuations compared to donepezil in AChE and tacrine in BuChE, but these remained within an acceptable range, demonstrating overall stable binding.

**Figure 5:**
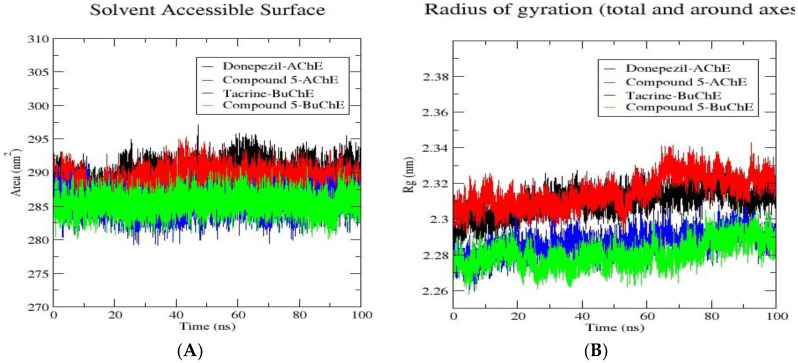
Structural dynamics of compound **5** bound to the 2 target proteins; SASA values (**A**) and Radius of Gyration of the target enzymes; AChE and BuChE in complex with compound **5** (**B**) calculated during the 100 ns of MD trajectories.

Further stability assessments using solvent-accessible surface area (SASA) and radius of gyration (Rg) analyses (Figure 5A,B) showed that both complexes remained compact throughout the simulations. The SASA fluctuated between 285 and 295 nm^2^ for AChE and 280 and 290 nm^2^ for BuChE, while the Rg plots indicated stable structural compactness.

**Figure 6:**
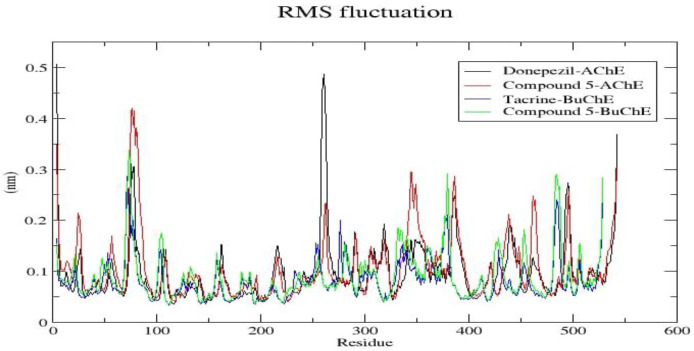
Root mean square fluctuation (RMSF) of compound **5** bound to the 2 target proteins, calculated during the 100 ns of MD trajectories.

**Figure 7:**
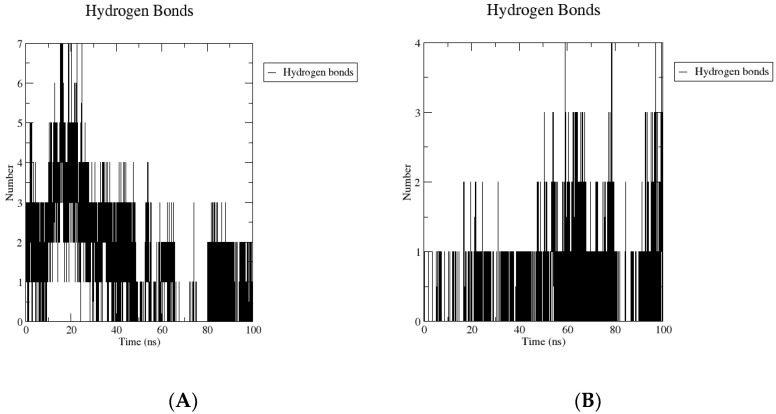
Intermolecular H-bonds of compound **5** bound to AChE (**A**) and BuChE (**B**) during the 100 ns of MD trajectories.

The root mean square fluctuation (RMSF) of backbone residues involved in ligand interactions (Figure 6) displayed minimal fluctuation (<0.2 nm) for key residues in both enzymes, corresponding to those identified in our docking study as critical for ligand interactions. For hydrogen bond analysis, compound **5** formed stable interactions with both AChE and BuChE. Specifically, in AChE, compound **5** initially formed 3–7 hydrogen bonds, which stabilized to 2–3 bonds during the middle of the simulation and maintained 2 stable bonds in the final 20 ns (Figure 7A). In contrast, donepezil’s hydrogen bonds decreased over time, indicating a reliance on hydrophobic interactions. For BuChE, compound **5** (Figure 7B) and tacrine both formed a stable single hydrogen bond, with the potential for 2–3 additional bonds at various points during the simulation. These findings suggest that compound **5** maintains strong and stable interactions with both AChE and BuChE, supported by consistent structural stability, minimal residue fluctuations, and robust hydrogen bonding throughout the simulations.

### 2.5. In Silico Physicochemical, Pharmacokinetic, and Drug-Likeness Data of ***5***, ***8***, and ***9*** Compared to Donepezil

The pharmacokinetics and pharmacodynamics, physicochemical properties, and drug-likeness of a molecule are all requirements for its consideration as a prospective therapeutic candidate. Therefore, Swiss ADME software (www.SwissADME.ch, accessed on 14 August 2023) was used to compare the silico ADME screening of compounds **5**, **8**, and **9** to that of donepezil. Compound **9** has the same high predicted gastrointestinal (GI) absorption as donepezil, as predicted by a boiled-egg model [73]. This means it may be able to be absorbed through the intestinal membrane.

Compounds **5** and **8** are also thought to be excreted out of the central nervous system by P-glycoprotein.

In addition, unlike donepezil, the target compounds **5**, **8**, and **9** exhibited a lack of BBB permeability, pointing to the fact that they may not reach the central nervous system (CNS) (Table 4). In addition, it was hypothesized that the compounds examined would not inhibit four of the major hepatic cytochrome P-450 (CYP) isoforms. Although, it is expected that they will inhibit just one of the hepatic CYP isoforms. Therefore, it should be given at different times than any other medicine the patient may be taking to prevent any adverse drug interactions. Donepezil has been predicted to inhibit CYP2D6 and CYP3A4 based on in silico data (Table 4). Although we did not conduct in vivo studies, this prediction is supported by existing literature confirming donepezil’s inhibition of both enzymes [74]. 

**Figure 8:**
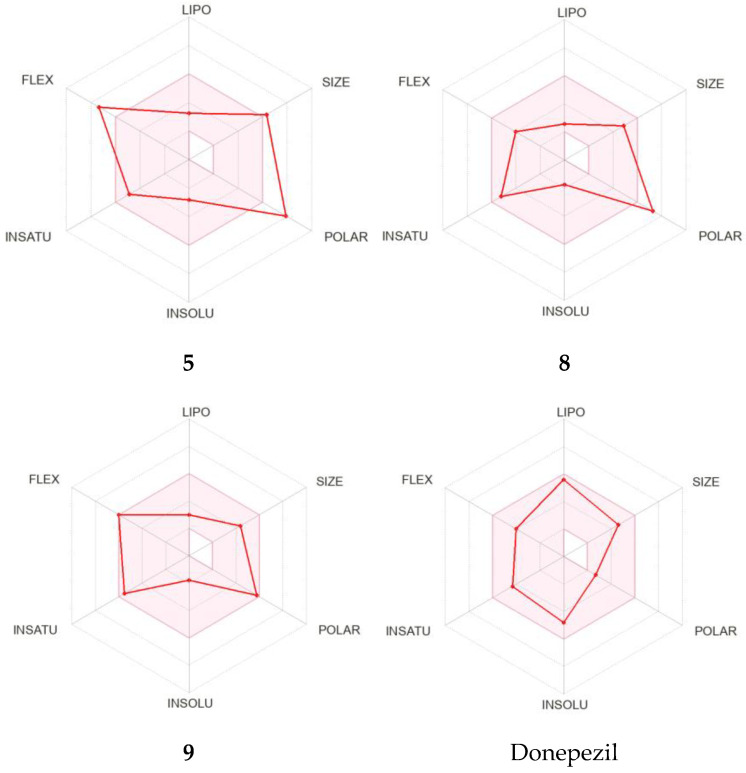
The bioavailability radar chart for the target compounds **5**, **8**, **9**, and donepezil (The pink zone indicates the oral bioavailability physicochemical space, while the red line reflects the oral bioavailability parameters).

The results of testing the oral bioavailability of **5**, **8**, **9**, and donepezil are shown in Figure 8. An oral bioavailability radar displays polarity (POLAR), size (SIZE), saturation (INSATU), flexibility (FLEX), solubility (INSOLU), and lipophilicity (LIPO) distribution. Lines in red depict the material’s calculated physicochemical qualities, whereas pink lines depict the best value for each parameter. All oral bioavailability markers for compound **9** were in pink, like donepezil. Except for the POLAR parameter, all the quantifiable physicochemical values for **8** fell inside the pink zone, indicating a favorable profile. Compound **5**, on the other hand, displayed a clear violation of the POLAR and FLEX criteria.

Compound **5** contains two ester groups, making it vulnerable to metabolism by esterases, enzymes that hydrolyze ester bonds. This could lead to the formation of inactive metabolites, reducing the compound’s stability and pharmacological efficacy, particularly in vivo, where esterases are abundant in tissues and blood. To address this, future studies could focus on modifying the ester groups to improve stability. Approaches include developing prodrugs to resist enzymatic hydrolysis or replacing ester groups with bioisosteres that retain similar properties but are less prone to esterase activity. These strategies may enhance compound **5**’s therapeutic potential and bioavailability.

### 2.6. In Silico Physicochemical Properites of ***5***, ***8***, and ***9*** Compared to Donepezil

Chemical and physical data for **5**, **8**, and **9** as well as donepezil, are provided in Table 5. Both **8** and **9**, the compounds of interest, have molecular weights of less than 500 Da, making them readily diffusible and absorbable across the cell membrane. Because they have an appropriate log *p*-value, **5**, **8**, and **9** are also likely to have good membrane permeability. In addition, it has been shown that compounds **8** and **9** are excellent hydrogen bond acceptors (9, 7) and hydrogen bond donors (3, 1), enabling the compound to pass across the water-filled voids of living-cell membranes. The fact that each of these compounds (**8** and **9**) has six or nine rotatable bonds, each of which is linked to a heavy atom, suggests that the molecules are very adaptable. In contrast, moderate TPSA was generated by the molecule’s polar atoms. The Lipinski rule of five states that medications with an M.wt. below 500, a log P below 5, an HBD below 5, and an HBA below 10 have acceptable absorption and bioavailability. In terms of their physical and chemical properties, substances **8** and **9** were quite like those of donepezil (Table 5).

### 2.7. Modeling Drug Action Using Pharmacophores

**Figure 9:**
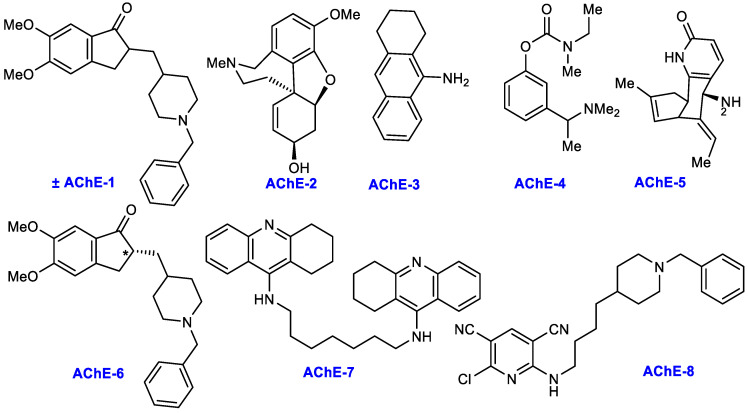
Chemical structures of AChE inhibitors in the training set.

The following steps outline the process of creating pharmacophores using the MOE 2015.10 program. Eight AChE inhibitors were employed as a training set for flexible alignment (Figure 9). The output of this flexible alignment includes a score (S) that indicates the quality of the configuration alignment; lower S values correspond to more favorable alignments. First, select the structure with the lowest S value in the alignment and copy it into the MOE window. Next, create a pharmacophore query in the pharmacophore query editor for the compounds included in the alignment training set. To validate the developed model against the entire dataset, conduct a pharmacophore search for compounds **5**, **8**, and **9**. The program utilizes the pharmacophore preprocessor, specifically implementing the PCH-All (Polarity–Charge–Hydrophobicity) system to label molecular conformations within the test set. After this, modify the query using the consensus query method to delve deeper into the structural information. Table 6 presents the relative mapping strengths from specific molecules to the generated hypothetical versions, quantified in terms of root mean square deviation (RMSD).

The following steps outline the process of creating pharmacophores. The MOE 2015.10 program employed the shown eight AChE inhibitors as a training set for flexible alignment (Figure 9). In the final output of the flexible alignment, you may access the data (S: Score of configuration alignment). It is possible that lower S values lead to more favorable alignments. Select the lowest S in the alignment structure and copy it into the MOE window. Create a pharmacophore query in the pharmacophore query editor for the compounds used in the alignment training set. To validate the developed model against the whole dataset, pharmacophore search is used (**5**, **8** and **9**). The program uses the pharmacophore preprocessor, with the PCH-All (Polarity–Charge–Hydrophobicity) implementation of a pharmacophore system for labeling test set molecular conformations. Then, modify the query using the consensus query method, and proceed to go even deeper into the information at hand. Table 6 displays relative mapping strengths from a specific molecule to a produced hypothetical version as provided by the computer in terms of root-mean-square deviation (RMSD).

### 2.8. Development of Pharmacophores

**Figure 10:**
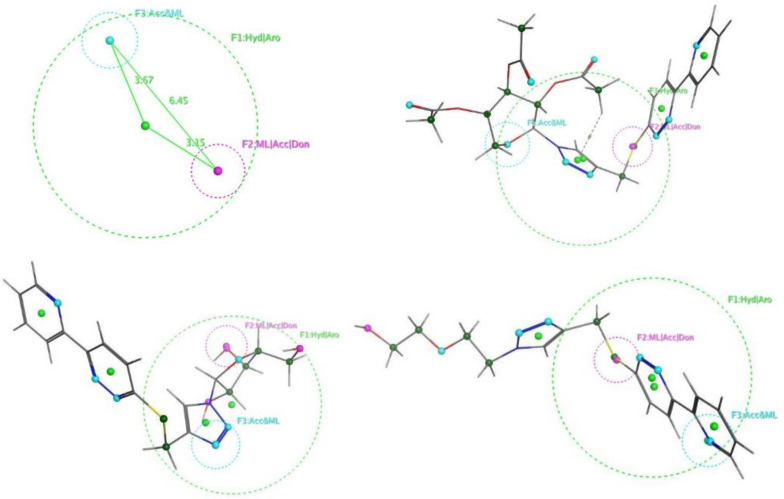
**Upper left panel**: Pharmacophore features and distances. **Upper right panel**: Superposition of **5** with the query. **Lower left panel**: Superposition of **8** with the query. **Lower right panel**: Superposition of **9** with the query.

The method’s goal is to create a pharmacophore model (hypothesis) from eight different AChE inhibitors and then test it [75]. Applications based on 3D pharmacophores typically include the following three steps: First, we need to build the three-dimensional structures of molecules from a training set that are known to have biological activity. After that, you obtain the pharmacophoric characteristics. Finally, we employ a strategy for searching databases to identify new compounds that possess the desired pharmacophoric features [76]. The root mean square deviation (RMSD) between the query and its ligand-target positions serves as an indicator of how well a compound fits the developed hypothetical model related to the molecule’s activity. Common pharmacophoric features include H-bond acceptors (Accs), donors (Dons), charged or ionizable groups (Cat and Ani), hydrophobic groups (Hyds), metal ligators (MLs), and/or aromatic rings (Aros). The preliminary results of the pharmacophoric analysis are presented in Table 6 and illustrated in Figure 10. With this revised consensus query, the test set returned three results **5**, **8**, and **9** (Figure 10). In Table 6, we see that an inhibitory effect becomes more powerful as the RMSD value decreases. Superimpositions of compound 5 showed the highest activity, with RMSD values of 0.1363. High inhibitory activity was also seen for compounds **8** and **9** (RMSD values of 0.2234 and 0.2468, respectively), indicating that the results of the in vitro bioassays are promising.

### 2.9. Structure–Activity Relationship (SAR)

The in vitro cholinesterase inhibition study and molecular docking analysis provide key insights into how structural modifications influence the activity of newly synthesized compounds against acetylcholinesterase (AChE) and butyrylcholinesterase (BuChE). Variations in sugar moieties, functional groups, and chain lengths across the compounds resulted in diverse inhibitory profiles. Docking studies, performed using AChE (PDB ID: 4EY7) and BuChE (PDB ID: 4BDS), revealed crucial binding interactions with the active sites, offering a molecular-level explanation for the in vitro results.

Compound **5**, with its protected acylated xylosyl moiety, emerged as the most potent inhibitor, displaying very low IC_50_ values for both AChE (0.266 µM) and BuChE (0.189 µM). This high potency was supported by docking results, showing a binding affinity score of −10.21 kcal/mol for AChE. Interactions included hydrogen bonding with Tyr 124, pi–pi stacking with Tyr 341, and multiple H–pi bonds with Tyr 337 and Trp 86, which likely contributed to its strong binding affinity. The compound also demonstrated an even higher binding score (−13.84 kcal/mol) for BuChE, further explaining its superior inhibitory capacity. Key interactions with His 448 and Tyr 332 reinforced its binding stability.

In comparison, compound **6**, the diastereomer of compound **5** with an acylated ribofuranosyl moiety, displayed much weaker inhibition (IC_50_ = 9.152 µM for AChE, 31.99 µM for BuChE) and a lower docking score (−6.35 kcal/mol). Despite multiple hydrogen bonds and pi–pi interactions with residues such as Trp 286, its stereochemistry appears to hinder effective alignment within the active site, highlighting the critical impact of stereochemistry on enzyme inhibition.

Compound **9**, with an -OCH_2_CH_2_OH side chain, demonstrated potent inhibition (AChE IC_50_ = 0.647 µM, BuChE IC_50_ = 0.384 µM), supported by its favorable docking score (−7.75 kcal/mol). Arene-H interactions with Val 294 and Phe 295 underscored the importance of this hydroxyl-containing chain in stabilizing its binding. Conversely, compound **10**, with a shorter -CH_2_CH_2_OH chain, exhibited weaker inhibition (AChE IC_50_ = 1.840 µM, BuChE IC_50_ = 6.06 µM). The docking score (−6.87 kcal/mol) reflected this reduced activity, suggesting that the shorter chain length and lower polarity weaken the binding interactions, thus diminishing inhibitory potency. This comparison emphasizes the role of chain length and polarity in optimizing enzyme binding.

When examining compound **11**, which contains a -CH_2_CH_2_NH_2_ substituent, both the in vitro (AChE IC_50_ = 14.63 µM, BuChE IC_50_ = 42.91 µM) and docking results (−6.35 kcal/mol) indicated it as the weakest inhibitor. Though pi–pi interactions with Tyr 341 and Tyr 337 were observed, the amine group (-NH2) likely introduces unfavorable steric and electronic effects, reducing binding efficacy.

A notable finding was the selective inhibition observed in compound **3**, which featured a protected acylated glucosyl moiety. It displayed significant inhibition of BuChE (IC_50_ = 0.297 µM) but much weaker activity against AChE (IC_50_ = 8.709 µM). Docking studies revealed key hydrogen bonds with Tyr 341 and Ser 293, as well as pi–pi interactions with Trp 286, potentially explaining the enhanced BuChE selectivity. The larger, more flexible active site of BuChE may favor hydrophobic and steric interactions with the acylated glucosyl moiety, whereas the more restrictive AChE active site limits its binding affinity.

In conclusion, molecular docking has reinforced the in vitro findings by illustrating the specific binding modalities and interactions that underpin cholinesterase inhibition. Compounds like **5** and **9** showed robust binding profiles due to extensive hydrogen bonding and pi–pi interactions with crucial amino acids, while compounds like **6** and **11** were less effective due to stereochemical constraints and less favorable functional group interactions. This combined approach offers valuable insights into the structural features necessary for designing more potent and selective cholinesterase inhibitors, with implications for developing therapeutic agents against neurodegenerative diseases.

## 3. Experimental Section

### 3.1. Synthetic Procedures

#### 3.1.1. Materials and Methods

All melting points are uncorrected and were measured using an Electro thermal IA 9100 apparatus. The ^1^H NMR and ^13^C NMR spectra were measured on a BRUKER 400 MHz for ^1^H NMR and 101 MHz for ^13^C NMR at National Research Center, Cairo, Egypt. The spectra were recorded by dissolving in CDCl_3_, using DMSO-d_6_ relative to tetramethylsilane (TMS) (0.00 ppm) as the standard reference. In ^1^H NMR, chemical shifts were reported in δ values using the internal standard (TMS) with a number of protons, multiplicities (s—singlet, d—doublet, t—triplet, q—quartet, and m—multiplet), and coupling constants (*J*) in hertz (Hz). The microanalytical data were carried out on a Vario El-Mentar instrument, at the Micro Analytical Laboratory, National Research Center, Cairo, Egypt. The reactions were monitored by thin layer chromatography (TLC). TLC was performed on Macherey–Nagel aluminum-backed plates, pre-coated with silica gel 60 (UV254). Column chromatography was carried out on silica gel 60 (0.040–0.063 mm) under flash conditions. All chemicals and solvents were purchased from Sigma-Aldrich, Alfa Aesar, and ACROS Organics and used as provided.

#### 3.1.2. General Synthetic Procedure 

##### Synthesis of Acetylated Sugars

Acetylation of sugars was performed according to literature reports by placing sugars in a 50 mL round-bottom flask with 20 mL of dichloromethane and a stirring bar. Then, acetic anhydride (1.2 eq. per OH) and perchloric acid (0.1 eq.) were added sequentially at 0 °C. The reaction mixture was stirred at the same temperature until TLC indicated the complete conversion of the starting material [68].

##### Synthesis of Pyranosyl Bromide [69]

In a dry round-bottom flask, (1 g) of acetylated sugar is dissolved in (10 mL) of dichloromethane under an inert atmosphere to prevent moisture interference. Hydrogen bromide gas is then added dropwise to the solution while stirring to facilitate the bromination reaction. Following this, 2.5 mL of a 33% *w/w* (45% *w/v*) solution of acetic acid is introduced dropwise at 0 °C to activate the bromination process. The mixture is then allowed to warm to room temperature and stirred for 2 hours. The reaction mixture is diluted with 50 mL of dichloromethane and washed successively with an 80 mL saturated aqueous solution of NaHCO₃. The organic layers are dried over sodium sulfate and concentrated to yield pyranosyl bromide.

##### Azidation of Aceto-Bromo Sugars

To a solution of pyranosyl bromide in dimethyl formamide (10 mL) was added sodium azide (1.2 eq), and the reaction mixture was allowed to stir at 50 °C for 1 h. After complete consumption of the starting material, the reaction mixture was extracted with ethyl acetate and washed with brine. The organic phase was dried over sodium sulfate, concentrated under reduced pressure, and purified by column chromatography using hexane and ethyl acetate (10–15%) as eluent [60].

##### Azidation of Alkyl Halides

To a round-bottom flask, halo-alkyl alcohol or amine (1 eq) and sodium azide (2.5 eq) in water (50 mL) were added. The mixture was stirred at 80 °C for 24 h and then cooled to room temperature. The solution was extracted with ethyl acetate (2 × 20 mL), and the organic layer was dried with sodium sulfate overnight and then filtered. After the removal of the solvent under vacuum, compound a was obtained as a crude liquid [69].

##### Pyridazinone Synthesis

2-acetylpyridine (1 eq) was added to cold solution of glyoxylic acid (1 eq) and potassium carbonate (2 eq) in water (100 mL), and the mixture was stirred at room temperature for 2.5 h and then cooled in ice. Acetic acid (7 eq) was added, followed by hydrazine (1.2 eq), and the stirred solution was heated under reflux for 2 h, then cooled into ice. Potassium carbonate was added to neutralize the solution, and the resultant precipitate was collected by filtration and washed with water, then i-PrOH. The isolated product appeared to be sufficiently clean, and no further purification was required [70].

##### Thionation of Pyridazinone

To a hot solution of pyridazinone (1 eq) in ethanol (20 mL), lawesson Reagent (0.8 eq) was added, and the reaction mixture was refluxed for 4 h. Then, the solvent was removed in vacuo, and the crude was treated with water and extracted with ethyl acetate. The organic layers were dried over anhydrous Na_2_SO_4_, filtered, and concentrated in vacuo. The residue was purified by flash chromatography on silica gel, using petroleum ether/ethyl acetate (from 7:3 to 4:6 *v*/*v*) as eluent to give a yellow solid [71].

##### Propagylation of Pyridazinethione

The pyridazinethione synthesized in the previous step was first dissolved in DMF (15 mL), and a sufficient amount of sodium hydride (2.5 eq) was added at 0 °C and stirred. One hour after addition, propargyl bromide (2 eq.) was added to the reaction. After completion of the reaction, the reaction mixture was extracted with ethyl acetate. The organic layers were passed through sodium sulfate and vacuum-dried. The product obtained was impure and was purified via a column, using hexane and ethyl acetate (45–50%) as eluent [70].

##### General Procedure for the Synthesis of Triazole-Linked Pyridazinethione Hybrids

Propargylated pyridazinethione, synthesized from a previous method (1 eq), was dissolved in acetonitrile (5 mL) in a round-bottom flask equipped with a magnetic stirring bar, then azides (1 eq) and copper Iodide (0.2 eq) were sequentially added to it and stirred at room temperature for 1 h. After completion of the reaction, as indicated by TLC, the reaction mixture was passed through a plug of celite, and excess iodine was quenched with sodium thiosulfate, extracted with ethyl acetate, and washed with brine. The organic phase was dried over sodium sulfate, concentrated under reduced pressure, and purified using column chromatography, using dichloromethane and methanol (5%) as eluent to obtain the analytically pure products.

##### Deacetylation of Synthesized Glycoconjugates Hybrids

Triazole-linked pyridazinethione glycoconjugates were dissolved in methanol (5 mL), sodium methoxide (0.1 eq) was added to it, and the reaction was allowed to stir at room temperature for 30 min. After complete deacetylation of the product, excess sodium methoxide was quenched with amberlite IR 120 resin. The solvent was evaporated under reduced pressure to obtain the desired products.

#### 3.1.3. Spectral Studies

##### 3-(Prop-2-yn-1-ylthio)-6-(pyridin-2-yl)pyridazine (**1**)

Prepared as described in general procedure 3.1.2.7 from 6-(pyridin-2-yl)pyridazine-3(2H)-thione (1.63 g, 8.65 mmol), sodium hydride (21.63 mmol, 0.52 g), and propargyl bromide (17.3 mmol, 2.06 g) to afford the **1** as a brown-colored powder in 83% yield, 1.63 g, m. p. 125–127 °C; IR (KBr, υ, cm^−1^): 3314, 3044, 3010, 2218. ^1^H NMR (DMSO-d^6^, δ ppm): 3.17 (s, 1H); 4.18 (s, 2H); 7.49 (t, *J* = 6.2 Hz, 1H); 7.80 (d, *J* = 9.5 Hz, 1H); 7.97 (t, *J* = 7.6 Hz, 1H); 8.34 (d, *J* = 8.6 Hz, 1H); 8.46 (d, *J* = 7.6 Hz, 1H); 8.69 (d, *J* = 3.85 Hz, 1H). ^13^C NMR (DMSO-d^6^, δ ppm): 18.5, 74.2, 80.3, 121.1, 124.6, 125.5, 127.0, 138.1, 150.2, 153.2, 156.0, 161.7. Anal. Calcd for C_12_H_9_N_3_S (227.29): C, 63.41; H, 3.99; N, 18.49; S, 14.11%. Found: C, 63.38; H, 4.02; N, 18.45; S, 14.15%.

##### (2R,3R,4S,5R,6R)-2-(Acetoxymethyl)-6-(4-(((6-(pyridin-2-yl)pyridazin-3-yl)thio)methyl)-1H-1,2,3-triazol-1-yl)tetrahydro-2H-pyran-3,4,5-triyltriacetate (**3**)

Prepared as described in general procedure 3.1.2.8 from 1-aza 2,3,4,6-tetra acetate glucopyranoside **2a** (100 mg, 0.267 mmol), propargylated pyridazinethione (0.267 mmol, 60.68 mg), and copper iodide (0.053 mmol, 10 mg) to afford the **3** as a dark brown powder in 70% yield, 112.25 mg, m. p. 108–110 °C; ^1^H NMR (MHz, CDCl_3_, δ ppm): 1.88, 2.04, 2.07, 2.08 (all s, 3H each, 4 × CH_3_CO); 4.01–4.05 (m, 1H); 4.16 (dd, *J* = 12.6, 1.8 Hz, 1H); 4.33 (dd, *J* = 12.6, 5.0 Hz, 1H); 4.80 (s, 2H); 5.27 (t, *J* = 9.6 Hz, 1H); 5.44 (d, *J* = 9.4 Hz, 1H); 5.52 (t, *J* = 9.4 Hz, 1H); 5.93 (d, *J* = 8.8 Hz, I H); 7.43 (t, *J* = 7.0 Hz, 1 H); 7.83 (d, *J* = 8.8 Hz, 1 H); 8.00 (s, 1H); 8.07 (t, *J* = 7.6 Hz, 1H); 8.36 (d, *J* = 8.4 Hz, 1H); 8.51 (d, *J* = 8 Hz, 1H); 8.79 (d, *J* = 4.0 Hz, 1H). ^13^C NMR (DMSO-d^6^, δ ppm): 20.3, 20.7, 20.8, 20.9, 36.3, 62.3, 68.1, 70.6, 72.7, 73.9, 84.4, 121.1, 123.2, 124.8, 126.0, 127.1, 138.1, 144.5, 150.0, 152.8, 156.7, 162.8, 168.9, 169.9, 170.1, 170.5. Anal. Calcd for C_26_H_28_N_6_O_9_S (600.60): C, 52.00; H, 4.70; N, 13.99; O, 23.97; S, 5.34%. Found: C, 51.92; H, 4.74; N, 14.05; O, 23.99; S, 5.30%.

##### (2R,3S,4S,5R,6R)-2-(Acetoxymethyl)-6-(4-(((6-(pyridin-2-yl)pyridazin-3-yl)thio)methyl)-1H-1,2,3-triazol-1-yl)tetrahydro-2H-pyran-3,4,5-triyl triacetate (**4**)

Prepared as described in general procedure 3.1.2.8 from 1-aza 2,3,4,6-tetra acetate galactopyranoside **2b** (100 mg, 0.267 mmol), propargylated pyridazinethione (0.267 mmol, 60.68 mg), and copper iodide (0.053 mmol, 10 mg) to afford the 4 as a dark brown powder in 73% yield, 117.0 mg, m. p. 112–114 °C; ^1^H NMR (CDCl_3_, δ ppm): 1.98, 2.05, 2.08, 2.16 (all s, 3H each, 4 × CH_3_CO); 4.00 (td, *J* = 6.8, 0.6 Hz, 1H); 4.16 (dd, *J* = 12.6, 1.8 Hz, 1H); 4.33 (dd, *J* = 12.6, 5.0 Hz, 1H); 4.80 (s, 2H); 5.28 (dd, *J* = 10.4, 3.6 Hz, 1H); 5.40 (dd, *J* = 10, 8 Hz, 1H); 5.52 (t, J = 9.2 Hz, 1H); 5.94 (d, J = 9.2 Hz, I H); 7.46 (t, J = 6.8 Hz, 1 H); 7.82 (d, *J* = 8.8 Hz, 1 H); 8.00 (s, 1H); 8.05 (t, *J* = 7.6 Hz, 1H); 8.29 (d, *J* = 8.4 Hz, 1H); 8.45 (d, *J* = 7.6 Hz, 1H); 8.69 (d, *J* = 3.6 Hz, 1H). ^13^C NMR (DMSO-d_6_, δ ppm): 20.4, 20.7, 20.8, 20.9, 36.3, 62.0, 67.9, 68.3, 70.9, 73.5, 84.8, 121.1, 123.6, 124.9, 126.1, 127.1, 138.4, 144.0, 150.1, 153.1, 156.2, 162.8, 168.90, 169.9, 170.4, 170.5. Anal. Calcd for C_26_H_28_N_6_O_9_S (600.60): C, 52.00; H, 4.70; N, 13.99; O, 23.97; S, 5.34%. Found: C, 51.94; H, 4.75; N, 14.07; O, 5.29; S, 23.99%.

##### (2S,3R,4S,5R)-2-(4-(((6-Phenylpyridazin-3-yl)thio)methyl)-1H-1,2,3-triazol-1-yl)tetrahydro-2H-pyran-3,4,5-triyl triacetate (**5**)

Prepared as described in general procedure 3.1.2.8 from 1-aza 2,3,4-tri acetate xylopyranoside **2c** (100 mg, 0.332 mmol), propargylated pyridazinethione (0.332 mmol, 75.46 mg), and copper iodide (0.066 mmol, 12.45 mg) to afford the **5** as a light brown powder in 65% yield, 114 mg, m. p. 128–130 °C; ^1^H NMR (CDCl_3_, δ ppm): 2.03, 2.04, 2.07 (all s, 3H each, 3 × CH_3_CO); 3.68 (dd, *J* = 11.6, 9.2 Hz, 1H); 4.30 (dd, *J* = 12.0, 5.6 Hz, 1H); 4.85 (s, 2H); 5.33 (t, *J* = 8.4 Hz, 1H); 5.40–5.46 (m, 1H); 5.63 (t, *J* = 8.8 Hz, 1H); 5.94 (d, *J* = 8.0 Hz, I H); 7.42 (t, *J* = 6.8 Hz, 1 H); 7.81 (d, *J* = 8.8 Hz, 1 H); 8.00 (s, 1H); 8.08 (t, *J* = 7.6 Hz, 1H); 8.37 (d, *J* = 8.0 Hz, 1H); 8.53 (d, *J* = 7.6 Hz, 1H); 8.79 (d, *J* = 4.0 Hz, 1H). ^13^C NMR (DMSO-d^6^, δ ppm): 20.0, 20.4, 20.5, 36.7, 67.9, 69.5, 70.8, 71.8, 86.2, 121.1, 123.3, 124.6, 125.5, 127.0, 138.1, 144.3, 150.2, 153.2, 156.0, 161.7, 169.7, 170.1, 170.5. Anal. Calcd for C_23_H_24_N_6_O_7_S (528.54): C, 52.27; H, 4.58; N, 15.90; O, 21.19; S, 6.07%. Found: C, 52.22; H, 4.61; N, 15.92; O, 21.23; S, 6.3%.

##### (2R,3R,4R,5R)-2-(Acetoxymethyl)-5-(4-(((6-(pyridin-2-yl)pyridazin-3-yl)thio)methyl)-1H-1,2,3-triazol-1-yl)tetrahydrofuran-3,4-diyl diacetate (**6**)

Prepared as described in general procedure 3.1.2.8 from 1-aza 2,3,4-tri acetate ribofuranoside **2d** (100 mg, 0.332 mmol), propargylated pyridazinethione (0.332 mmol, 75.46 mg), and copper iodide (0.066 mmol, 12.45 mg) to afford the **6** as a black powder in 65% yield, 105.25 mg, m. p. 96–98 °C; ^1^H NMR (CDCl_3_, δ ppm): 2.02 (s, 3H, COCH_3_); 2.10 (s, 6H each, 2 × CH_3_CO); 4.23 (dd, *J* = 12.4, 4.0 Hz, 1H); 4.40 (dd, *J* = 12.4, 2.8 Hz, 1H); 4.48 (dd, *J* = 8.2, 4.2, 1H); 4.80 (s, 2H); 5.52 (t, *J* = 5.4 Hz, 1H); 5.9 (dd, *J* = 4.8, 4.0, 1H); 6.18 (d, *J* = 3.6 Hz, 1H); 7.41 (t, *J* = 6.8 Hz, 1 H); 7.80 (d, *J* = 8.8 Hz, 1 H); 7.99 (s, 1H); 8.06 (t, *J* = 7.6 Hz, 1H); 8.34 (d, *J* = 8.4 Hz, 1H); 8.50 (d, *J* = 7.6 Hz, 1H); 8.77 (d, *J* = 3.6 Hz, 1H). ^13^C NMR (DMSO-d^6^, δ ppm): 20.3, 20.7, 20.8, 36.2, 62.3, 70.6, 72.7, 74.8, 84.4, 121.2, 123.2, 124.8, 126.0, 127.1, 138.2, 144.5, 150.0, 152.8, 156.7, 162.9, 168.9, 169.9, 170.1. Anal. Calcd for C_23_H_24_N_6_O_7_S (528.54): C, 52.27; H, 4.58; N, 15.90; O, 21.19; S, 6.07%. Found: C, 52.19; H, 4.61; N, 15.95; O, 21.22; S, 6.03%.

##### (2R,3S,4S,5R,6R)-2-(Hydroxymethyl)-6-(4-(((6-(pyridin-2-yl)pyridazin-3-yl)thio)methyl)-1H-1,2,3-triazol-1-yl)tetrahydro-2H-pyran-3,4,5-triol (**7**)

Prepared as described in general procedure 3.1.2.9 from **3** (49.25 mg, 0.082 mmol), NaOMe (0.016 mmol, 1 mg) to afford the **7** as a brown powder in 89% yield, 31.55 mg, m. p. 96–98 °C; IR (KBr, υ, cm^−1^): 3422–3380, 3040, 3010; 2980. ^1^H NMR (DMSO-d_6_, δ ppm): 3.23 (t, *J* = 8.8 Hz, 1H); 3.40–3.50 (m, 3H); 3.63–3.74 (m, 2H); 4.66 (d, *J* = 5.6 Hz, 1H, OH); 4.73 (t, *J* = 5.6 Hz, 1H, OH); 4.81 (s, 2H); 5.04 (d, *J* = 5.6 Hz, 1 H, OH); 5.23 (d, *J* = 6 Hz, 1H, OH); 5.64 (d, *J* = 8.8 Hz, 1H); 7.46 (t, *J* = 6.8 Hz, 1 H); 7.84 (d, *J* = 8.8 Hz, 1 H); 8.01 (s, 1H); 8.08 (t, *J* = 7.6 Hz, 1H); 8.39 (d, *J* = 8.0 Hz, 1H); 8.56 (d, *J* = 7.6 Hz, 1H); 8.80 (d, *J* = 3.6 Hz, 1H). ^13^C NMR (DMSO-d^6^, δ ppm): 36.3, 60.9, 71.1, 72.3, 76.9, 80.0, 86.7, 121.2, 123.9, 124.9, 126.0, 127.1, 138.4, 144.0, 150.0, 153.2, 156.2, 162.9. Anal. Calcd for C_18_H_20_N_6_O_5_S (432.46): C, 49.99; H, 4.66; N, 19.43; O, 18.50; S, 7.41%. Found: C, 50.05; H, 4.59; N, 19.45; O, 18.44; S, 7.47%.

##### (2R,3S,4R,5R)-2-(Hydroxymethyl)-5-(4-(((6-(pyridin-2-yl)pyridazin-3-yl)thio)methyl)-1H-1,2,3-triazol-1-yl)tetrahydrofuran-3,4-diol (**8**)

Prepared as described in general procedure 3.1.2.9 from **6** (43.34 mg, 0.082 mmol), NaOMe (0.016 mmol, 1 mg) to afford the **8** as a black powder in 88% yield, 29 mg, m. p. 92–94 °C; IR (KBr, υ, cm^−1^): 3418–3398, 3046, 3014, 2987; ^1^H NMR (DMSO-d_6_, δ ppm): 3.46–3.51 (m, 1H); 3.55–3.61 (m, 1H); 3.94 (dd, *J* = 8.0, 4.0 Hz, 1H); 4.10 (dd, *J* = 9.4, 4.8 Hz, 1H); 4.32 (dd, *J* = 10.6, 4.8 Hz, 1H); 4.79 (s, 2H); 4.98 (t, *J* = 5.2 Hz, 1H, OH); 5.21 (d, *J* = 5.2 Hz, I H, OH); 5.51 (d, *J* = 6.4, 1H, OH); 5.80 (d, *J* = 4.8, 1H); 7.46 (t, *J* = 6.8 Hz, 1 H); 7.85 (d, *J* = 8.8 Hz, 1 H); 8.02 (s, 1H); 8.10 (t, *J* = 7.6 Hz, 1H); 8.39 (d, *J* = 8.4 Hz, 1H); 8.56 (d, *J* = 7.6 Hz, 1H); 8.80 (d, *J* = 3.6 Hz, 1H). ^13^C NMR (DMSO-d_6_, δ ppm): 36.3, 61.8, 70.8, 74.1, 79.2, 87.2, 120.9, 123.7, 124.8, 126.2, 127.4, 138.0, 144.0, 150.1, 153.1, 156.1, 162.9. Anal. Calcd for C_17_H_18_N_6_O_4_S (402.43): C, 50.74; H, 4.51; N, 20.88; O, 15.90; S, 7.97%. Found: C, 50.66; H, 4.54; N, 20.93; O, 15.94; S, 7.93%.

##### 2-(2-(4-(((6-Pyridin-2-yl)pyridazin-3-yl)thio)methyl)-1H-1,2,3-triazol-1-yl)ethoxy)ethan-1-ol (**9**)

Prepared as described in general procedure 3.1.2.8 from 2-(2-azidoethoxy)ethan-1-ol **2e** (87.20 mg, 0.665 mmol), propargylated pyridazinethione (0.665 mmol, 148.87 mg), and copper iodide (0.132 mmol, 24.94mg) to afford the **9** as a black powder in 72% yield, 171.60 mg, m. p. 116–118 °C; ^1^H NMR (CDCl_3_, δ ppm): 3.51 (t, *J* = 4.4 Hz, 2H); 3.63 (t, *J* = 4.8 Hz, 2H); 3.87 (t, *J* = 5.2 Hz, 2H); 4.59 (t, *J* = 5.2 Hz, 2H); 4.83 (s, 2H); 4.96 (brs, 1H, OH); 7.43 (t, *J* = 6.8 Hz, 1 H); 7.81 (d, *J* = 8.8 Hz, 1 H); 8.00 (s, 1H); 8.06 (t, *J* = 7.6 Hz, 1H); 8.35 (d, *J* = 8.4 Hz, 1H); 8.52 (d, *J* = 7.6 Hz, 1H); 8.78 (d, *J* = 3.6 Hz, 1H). ^13^C NMR (DMSO-d^6^, δ ppm): 36.2, 51.4, 62.0, 70.3, 73.5, 121.1, 123.2, 124.6, 125.5, 127.0, 138.14, 144.3, 150.2, 153.2, 156.0, 161.7. Anal. Calcd for C_16_H_18_N_6_O_2_S (358.42): C, 53.62; H, 5.06; N, 23.45; O, 8.93; S, 8.94%. Found: C, 53.70; H, 4.98; N, 23.47; O, 8.97; S, 8.88%.

##### 2-(4-(((6-(Pyridin-2-yl)pyridazin-3-yl)thio)methyl)-1H-1,2,3-triazol-1-yl)ethan-1-ol (**10**)

Prepared as described in general procedure 3.1.2.8 from 2-azidoethan-1-ol **2f** (57.90 mg, 0.665 mmol), propargylated pyridazinethione (0.665 mmol, 148.87 mg), and copper iodide (0.132 mmol, 24.94mg) to afford the **10** as a black powder in 69% yield, 144.25 mg, m. p. 112–114 °C; ^1^H NMR (CDCl_3_, δ ppm): 4.02 (t, *J* = 5.2 Hz, 2H); 4.39 (brs, 1H, OH); 4.55 (t, *J* = 5.2, 2H); 4.78 (s, 2H); 7.44 (t, *J* = 6.8 Hz, 1 H); 7.81 (d, *J* = 8.8 Hz, 1 H); 7.97 (s, 1H); 8.08 (t, *J* = 7.6 Hz, 1H); 8.33 (d, *J* = 8.4 Hz, 1H); 8.53 (d, *J* = 7.6 Hz, 1H); 8.74 (d, *J* = 4.0 Hz, 1H). ^13^C NMR (DMSO-d_6_, δ ppm): 36.4, 52.9, 61.2, 120.9, 123.6, 124.8, 126.2, 127.5, 138.9, 144.0, 151.0, 153.0, 156.1, 162.8. Anal. Calcd for C_14_H_14_N_6_OS (314.37): C, 53.49; H, 4.49; N, 26.73; O, 5.09; S, 10.20%. Found: C, 53.51; H, 4.52; N, 26.67; O, 5.12; S, 10.18%.

##### 2-(4-(((6-(Pyridin-2-yl)pyridazin-3-yl)thio)methyl)-1H-1,2,3-triazol-1-yl)ethan-1-amine (**11**) 

Prepared as described in general procedure 3.1.2.8 from 2-azidoethan-1-amine **2g** (57.26 mg, 0.665 mmol), propargylated pyridazinethione (0.665 mmol, 148.87 mg), and copper iodide (0.132 mmol, 24.94mg) to afford the **11** as a dark brown powder in 65% yield, 135.45 mg, m. p. 105–107 °C; ^1^H NMR (CDCl_3_, δ ppm): 3.12 (brs, 2H, NH2); 3.20 (t, *J* = 5.2 Hz, 2H); 4.55 (t, *J* = 5.2 Hz, 2H); 4.82 (s, 2H); 7.42 (t, *J* = 6.8 Hz, 1 H); 7.81 (d, *J* = 8.8 Hz, 1 H); 8.00 (s, 1H); 8.07 (t, *J* = 7.6 Hz, 1H); 8.35 (d, *J* = 8.0 Hz, 1H); 8.51 (d, *J* = 7.6 Hz, 1H); 8.78 (d, *J* = 3.6 Hz, 1H). ^13^C NMR (DMSO-d_6_, δ ppm): 37.1, 42.1, 52.7, 121.1, 123.2, 124.6, 125.5, 127.0, 138.1, 144.3, 150.2, 153.2, 156.0, 162.7. Anal. Calcd for C_14_H_15_N_7_S (313.38): C, 53.66; H, 4.82; N, 31.29; S, 10.23%. Found: C, 53.63; H, 4.85; N, 31.26; S, 10.26%.

### 3.2. Biology

#### In Vitro Cholinesterases Inhibition and Selectivity Assay

A QuantiChrom™ Acetylcholinesterase Inhibitor Screening Kit (IACE-100) and Butyrylcholinesterase Activity Kit (Catalog # EIABCHEF (192 tests)) were sourced from BioAssay Systems, Hayward, CA, United States, and were used to perform acetylcholinesterase enzyme inhibition assays. Serial logarithmic dilutions (concentrations from 0.01 to 100 µM) were prepared for the target compounds **1** and **3**–**11**, in addition to donepezil, tacrine, and rivastigmine as positive controls. Experimental procedures were followed as instructed by the kits’ manufacturer. The enzymes used in the assays were of human origin. IC_50_ values of the compounds were calculated from the obtained dose response curve, and selectivity index was calculated as a ratio between (AChE/BuChE) IC_50_.

### 3.3. Molecular Docking of Compound ***5*** against Acetylcholinesterase and Butyrylcholinesterase

Molecular docking is a powerful computational technique used to evaluate the potential energetic and geometric fit of a ligand within the active site of a protein. Gaussian 09 was utilized to generate files containing the structures of the compounds in PDB format. The structures of Acetylcholinesterase (AChE) and Butyrylcholinesterase (BuChE) were retrieved from the Protein Data Bank (PDB IDs: 4EY7 and 4BDS, respectively). Molecular docking analyses were performed using MOE 2015. The original enzyme structures, along with their co-crystallized ligands, were re-docked using the program’s default settings. The binding energy (in kcal/mol) and binding distances (in Å) for the amino acid interactions are presented in Table 2.

### 3.4. In Silico Pharmacokinetics and Toxicity Profile Prediction

The pharmacokinetics and pharmacodynamics, physicochemical properties, and drug-likeness of a molecule are all requirements for its consideration as a prospective therapeutic candidate. Therefore, Swiss ADME software (www.SwissADME.ch, accessed on 14 August 2023) was used to compare the silico ADME screening of compounds **5**, **8**, and **9** to that of donepezil. 

### 3.5. Molecular Dynamics Simulation

Finally, to investigate the stability of the best hit (**5**) binding to the TWO target proteins, AChE and BuChE enzymes pockets (PDB IDs 4EY7 and 1BDS, respectively), we conducted thorough molecular dynamics (MD) simulations using GROMACS-2023.1. The most favorable binding pose of compound **5** was subjected to a 100-nanosecond (ns) simulation while bound to the AChE ligand-binding site. For generating protein topology, we utilized the CHARMM36 force field [77], and the ligand topology was generated using a General force field (CGenFF) server [78]. Solvation was achieved employing a dodecahedral unit cell with periodic boundary conditions set at 10 Å to confine atom interactions within the simulation box. Protein neutralization was accomplished by introducing sodium and chloride ions using the steepest descent minimization algorithm. Energy minimization was performed to alleviate steric clashes, with a force cutoff of 10.0 kJ/mol and a maximum of 50,000 steps. Subsequently, NVT (canonical ensemble) and NPT (isothermal–isobaric ensemble) equilibration processes were conducted for 50,000 steps each, equivalent to 10 picoseconds, employing a modified Berendsen thermostat and leap-frog integrator. The MD simulations were then carried out for 100 ns, using a time step of 2 femtoseconds per step.

## 4. Conclusions

Using the click chemistry approach, this study explores the development and evaluation of novel pyridazine-containing compounds as potential inhibitors of acetylcholinesterase (AChE) and butyrylcholinesterase (BuChE). Among these, compound **5** emerges as the most promising inhibitor, displaying IC_50_ values of 0.26 µM for AChE and 0.18 µM for BuChE. Biological investigations confirm compound **5**’s superior potency, outperforming rivastigmine and tacrine, while showing competitive results against donepezil. Docking studies reveal that compound **5** exhibits the highest binding affinity (−10.21 kcal/mol) to AChE, forming multiple stable interactions, including hydrogen bonds and π–π stacking. Similar results are observed with BuChE, where compound **5** shows a high binding affinity of −13.84 kcal/mol. Molecular dynamics simulations further support these findings, indicating stable interactions and minimal fluctuations, reinforcing compound **5**’s potential as a strong inhibitor. Pharmacophore modeling highlights the key features necessary for effective inhibition. Compounds **5**, **8**, and **9**, identified through in silico ADME studies, demonstrate favorable pharmacokinetic properties, with compound **5** showing the highest activity based on RMSD values. The structure–activity relationship (SAR) analysis underscores the impact of specific substituents on inhibitory activity. Compound **5**, with its acylated xylosyl moiety, stands out as the most effective, followed by compound **9** with -OCH₂CH₂OH and compound **8** with ribofuranosyl groups. In summary, compound **5** shows exceptional promise for Alzheimer’s disease treatment through effective cholinesterase inhibition, supported by robust in silico and in vitro analyses. This research provides a solid foundation for the further optimization and development of these novel inhibitors.

## Figures and Tables

**Table 1:** IC_50_ values of compounds **1**, **3**–**11**, donepezil, tacrine, and rivastigmine against acetylcholinesterase (AChE) and butyrylcholinesterase (BuChE).

Compounds	AChE (µM)	BuChE (µM)	Selectivity Index (AChE/BuChE)
**1**	11.54 ± 0.48	4.37 ± 0.14	2.64
**3**	8.70 ± 0.36	0.29 ± 0.01	29.32
**4**	11.86 ± 0.50	14.63 ± 0.48	0.81
**5**	0.26 ± 0.01	0.18 ± 0.01	1.41
**6**	9.15 ± 0.38	31.99 ± 1.05	0.29
**7**	3.14 ± 0.13	7.90 ± 0.26	0.39
**8**	0.64 ± 0.02	5.35 ± 0.17	0.12
**9**	0.64 ± 0.02	0.38 ± 0.01	1.68
**10**	1.84 ± 0.07	6.06 ± 0.20	0.30
**11**	14.63 ± 0.61	42.91 ± 1.52	0.34
Donepezil	0.17 ± 0.00	0.41 ± 0.01	0.43
Tacrine	0.44 ± 0.02	0.12 ± 0.01	3.47
Rivastigmine	2.76 ± 0.11	18.08 ± 0.59	0.15

**Table 2:** Docking-interaction data of compounds **1**, **3**–**11** and donepezil inside AChE (PDB ID: 4EY7) active site.

Compound	Binding Affinity (Kcal/mol)	Affinity Bond Strength (Kcal/mol)	Affinity Bond Length (in A° from the Main Residue)	Amino Acids	Ligand	Interaction
**1**	−6.35	−0.0	3.70	TRP 286	6-ring	pi–pi
		−0.0	3.93	TYR 341	6-ring	pi–pi
**3**	−7.28	−1.4	3.34	TYR 341	C 29	H–donor
		−1.8	3.20	SER 293	O 51	H–acceptor
		−0.7	3.90	TYR 337	C 15	H–pi
		−0.0	3.95	TRP 286	5-ring	pi–pi
**4**	−7.47	−1.1	3.17	ASP 74	C 40	H–donor
		−0.6	3.52	TYR 337	C 67	H–pi
**5**	−10.21	−1.1	2.73	TYR 124	O 56	H–acceptor
		−0.9	3.92	TYR 337	C 37	H–pi
		−0.6	4.17	TRP 86	C 44	H–pi
		−0.0	3.83	TYR 341	5-ring	pi–pi
**6**	−9.28	−1.5	2.82	GLY 121	O 42	H–acceptor
		−1.3	2.81	GLY 122	O 42	H–acceptor
		−0.8	3.12	TYR 124	O 49	H–acceptor
		−0.6	4.32	TYR 341	C 6	H–pi
		−0.7	4.73	TRP 286	6-ring	pi–H
		−0.0	3.80	TRP 286	6-ring	pi–pi
**7**	−7.62	−0.9	3.22	GLY 120	O 43	H–donor
		−2.6	2.78	HIS 447	O 49	H–donor
		−0.0	3.99	TYR 341	6-ring	pi–pi
**8**	−8.26	−1.2	2.82	GLY 120	O 41	H–donor
		−1.4	3.04	GLU 202	O 43	H–donor
		−2.3	3.08	GLU 202	O 45	H–donor
		−0.6	4.43	GLY 121	5-ring	pi–H
		−0.0	3.55	TYR 341	6-ring	pi–pi
**9**	−7.75	−0.6	3.90	VAL 294	6-ring	pi–H
		−1.0	4.60	PHE 295	6-ring	pi–H
**10**	−7.27	−1.1	2.98	GLU 202	O 35	H–donor
		−1.0	4.02	TYR 337	C 3	H–pi
		−0.6	3.71	TRP 86	C 29	H–pi
		−1.3	3.81	HIS 447	5-ring	pi–H
		−0.0	3.78	TYR 341	6-ring	pi–pi
**11**	−7.09	−1.6	3.61	ARG 296	N 10	H–acceptor
		−0.0	3.62	TYR 341	6-ring	pi–pi
		−0.0	3.87	TYR 337	6-ring	pi–pi
Donepezil	−8.65	−0.9	3.66	TRP 286	C 17	H–pi
		−0.6	3.55	TYR 341	C 26	H–pi
		−0.0	3.68	TRP 286	6-ring	pi–pi

**Table 3:** Docking interaction data calculation of compounds **5** and tacrine with BuChE (PDB ID: 4BDS) active spot.

Compound	Binding Energy (Kcal/mol)	Bond Strength (Kcal/mol)	Bond Length (in A° from the Main Residue)	Amino Acids	Ligand	Interaction
**5**	−13.84	−2.2	3.37	HIS 438	S 18	Donor
		−0.0	3.93	TYR 332	6-ring	pi–pi
Tacrine	−7.45	−0.7	4.06	TRP 82	C 13	H–pi
		−0.0	3.58	TRP 82	6-ring	pi–pi

**Table 4:** The in silico-predicted pharmacokinetics for compounds **5**, **8**, **9**, and donepezil.

Molecule	GI Absorption	BBB Permeant	Pgp Substrate	CYP1A2 Inhibitor	CYP2C19 Inhibitor	CYP2C9 Inhibitor	CYP2D6 Inhibitor	CYP3A4 Inhibitor
**5**	Low	No	No	No	No	No	No	Yes
**8**	Low	No	No	No	No	No	No	Yes
**9**	High	No	No	No	No	No	No	Yes
Donepezil	High	Yes	Yes	No	No	No	Yes	Yes

**Table 5:** In silico physicochemical properties for compounds **5**, **8**, **9**, and donepezil.

Molecule	MW ≤500 ^a^	Log P_o/w_ ≤ 5 ^b^	HBA ≤10 ^c^	HBD ≤5 ^d^	TPSA Ǻ^2^ <160 ^e^	NRB ≤10 ^f^	#Heavy Atoms	Lipinski #Violations
**5**	528.54	3.3	12	0	182.81	11	37	2
**8**	402.43	2.57	9	3	164.6	6	28	0
**9**	358.42	2.95	7	1	124.14	9	25	0
Donepezil	379.49	3.92	4	0	38.77	6	28	0

^a^ MW, molecular weight; ^b^ Log P_o/w_, partition coefficient octanol/water; ^c^ HBA, number of H-bond acceptors; ^d^ HBD, number of H-bond donors; ^e^ TPSA, topological polar surface area; ^f^ NRB, number of rotatable bonds.

**Table 6:** Training inhibitor pharmacophoric and structural characteristics and RMSD values for the hit set.

Pharmacophoric Features	Structure Features	Compound	RMSD
F1: Hyd/Aro F2: ML/Acc/Don F3: Acc&ML	five heterocycles (triazole ring, tetrahydrofuran), six heterocycles (pyridazine ring), oxygen of pyran, -CH_3_, OH, -CH_2_. OH, -S-CH_2_. oxygen of pyran ring, nitrogen of triazole and pyridine ring.	**5**	0.1363
		**8**	0.2234
		**9**	0.2468

## Data Availability

The data presented in this study are available.

## Supplementary Materials

The following supporting information can be downloaded at: https://www.mdpi.com/article/10.3390/ph17101407/s1, See Figures S1–S20 and Table S1 in the Supplementary Materials.
