# Supplementary material for: Novel Dual Acetyl- and Butyrylcholinesterase Inhibitors Based on the Pyridyl–Pyridazine Moiety for the Potential Treatment of Alzheimer’s Disease
Source: Pharmaceuticals (Basel). 2024 Oct 21;17(10):1407. doi: 10.3390/ph17101407 (PMC11510214; doi:10.3390/ph17101407)
Supplement: Supplementary file 1 [file pharmaceuticals-17-01407-s001.zip › pharmaceuticals-3221849-supplementary.pdf]

Supporting Information

Novel Dual Acetyl- and Butyrylcholinesterase Inhibitors Based on the Pyridyl-Pyridazine Moiety for the Potential Treatment of Alzheimer's Disease

^{1}H-NMR and ^{13}C-NMR of all compounds

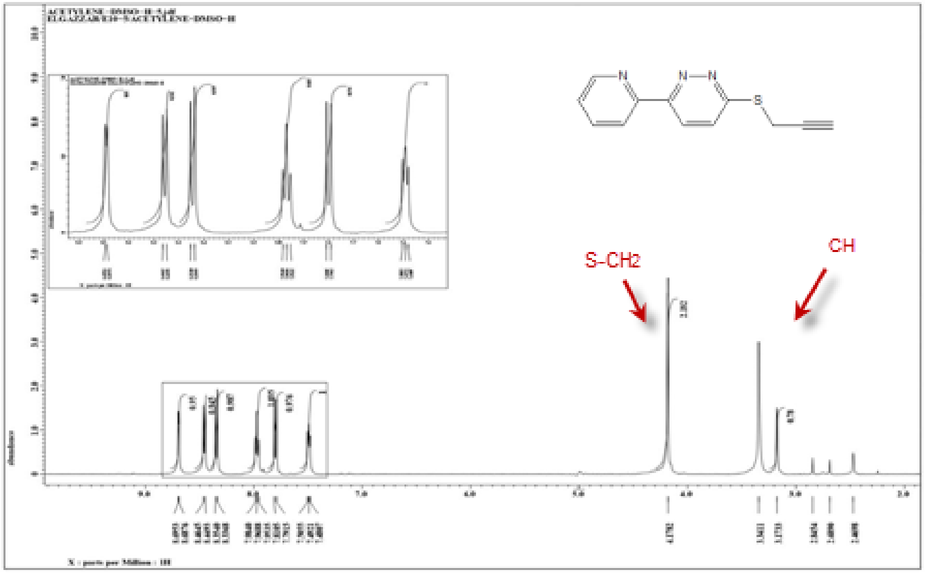

Figure S1: ^{1}H-NMR of Compound **1**

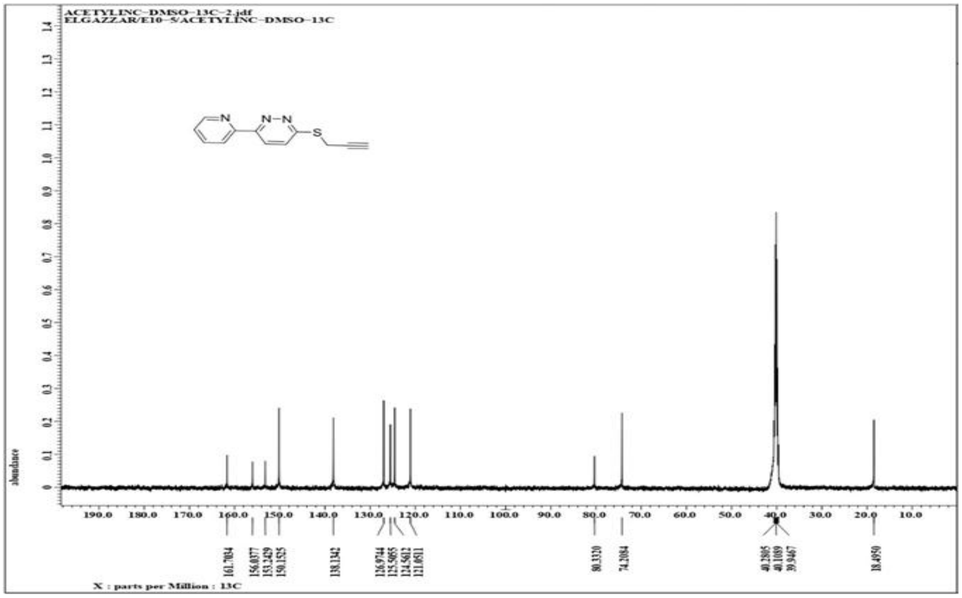

Figure S2: ^{13}C-NMR of Compound **1**

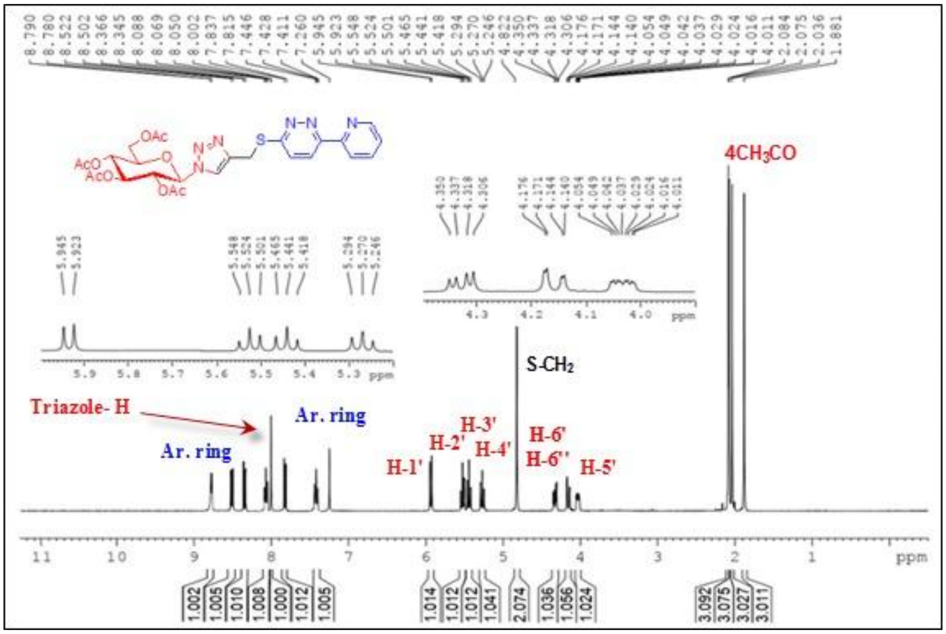

Figure S3: ^{1}H-NMR of Compound **3**

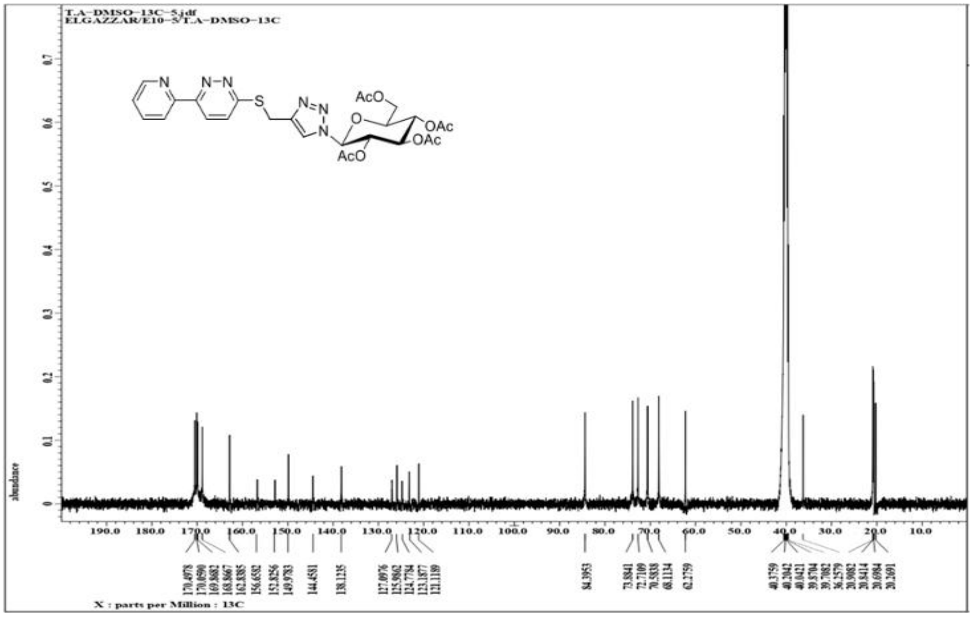

Figure S4: ^{13}C-NMR of Compound **3**

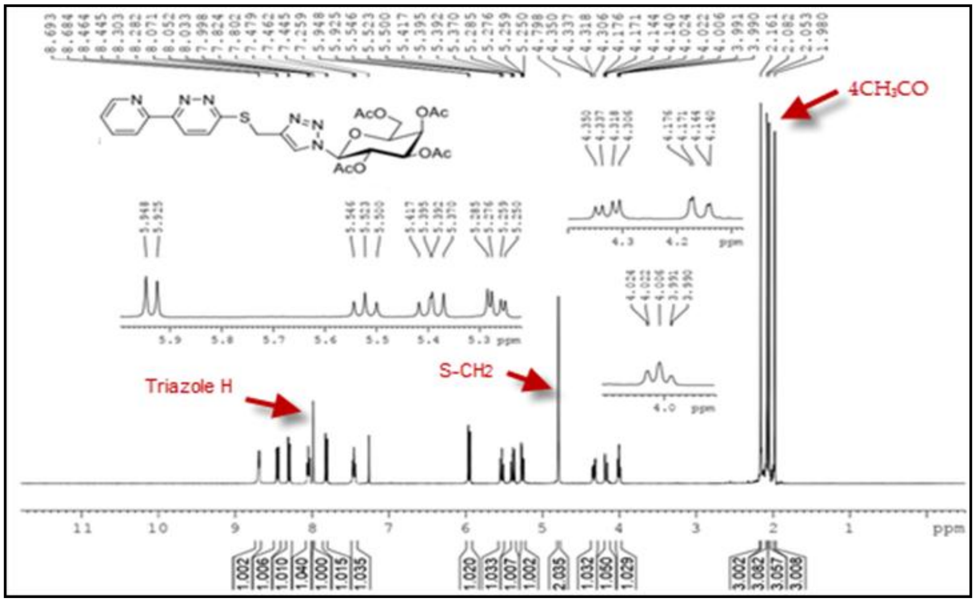

Figure S5: ^{1}H-NMR of Compound **4**

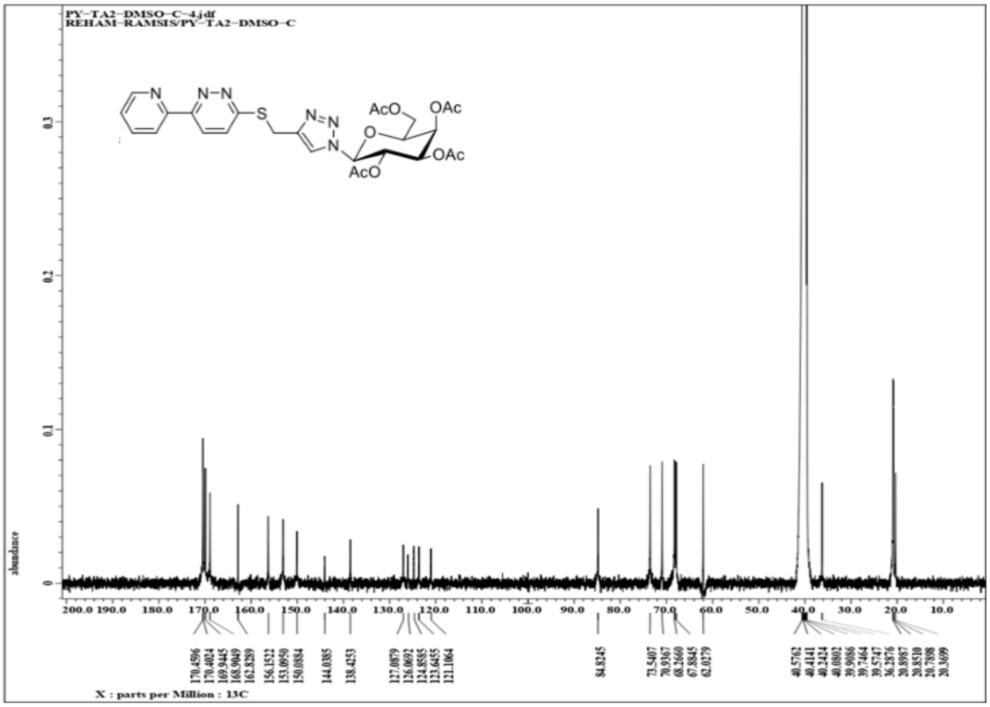

Figure S6: ^{13}C-NMR of Compound **4**

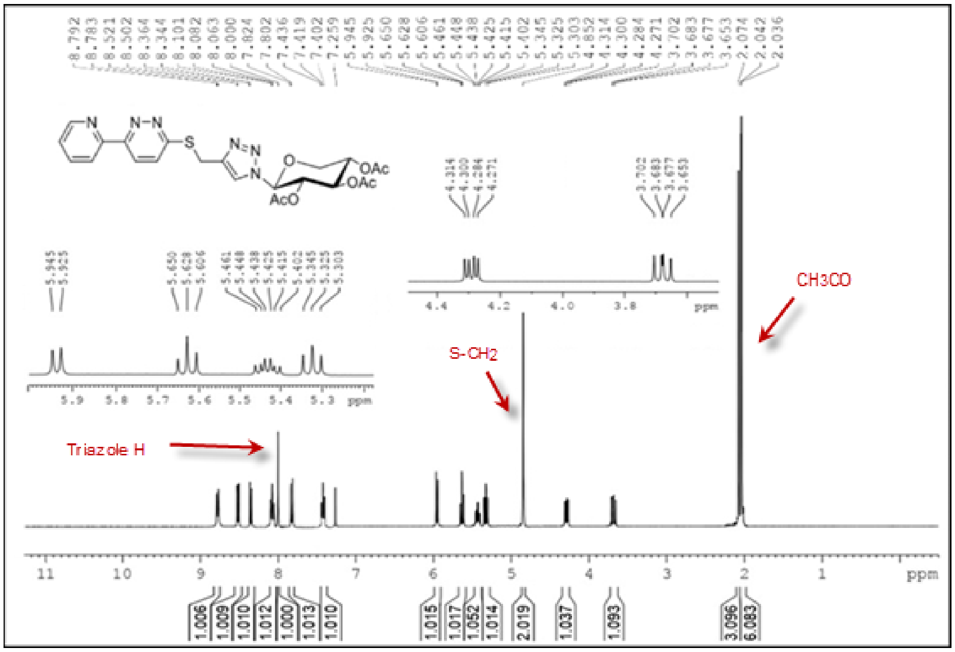

Figure S7: ^{1}H-NMR of Compound **5**

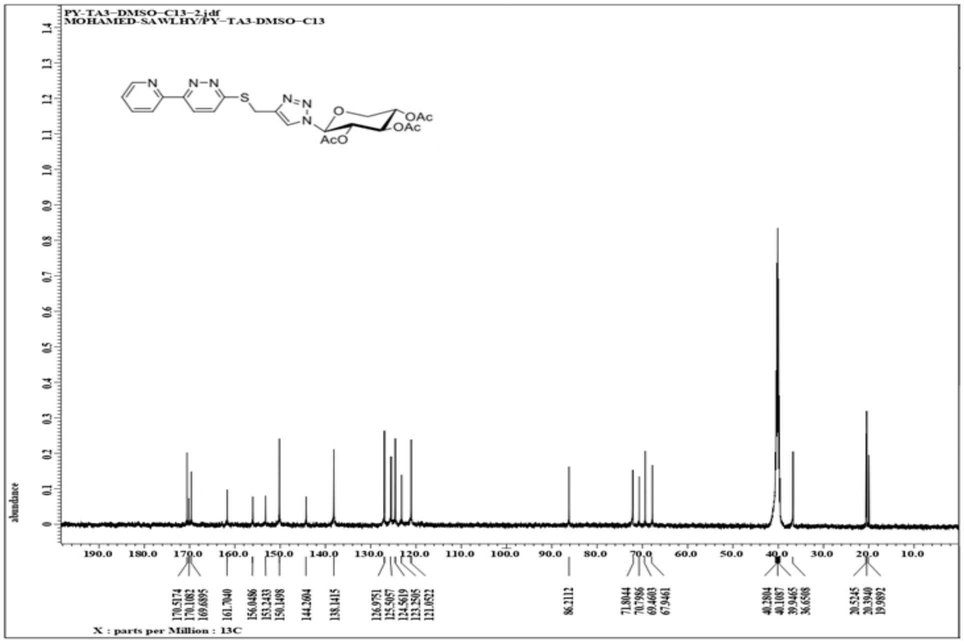

Figure S8: ^{13}C-NMR of Compound **5**

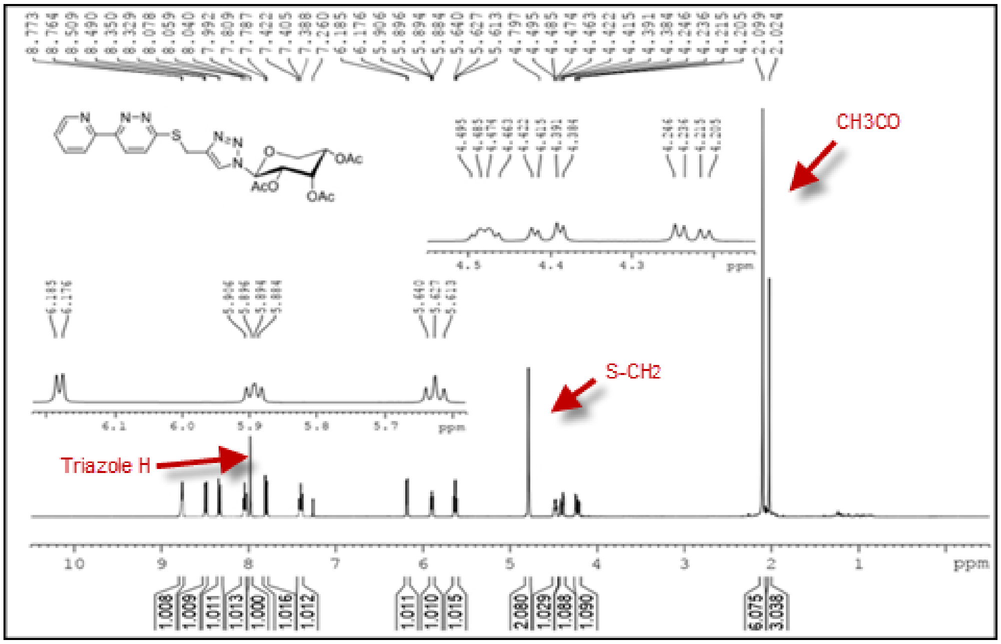

Figure S9: ^{1}H-NMR of Compound **6**

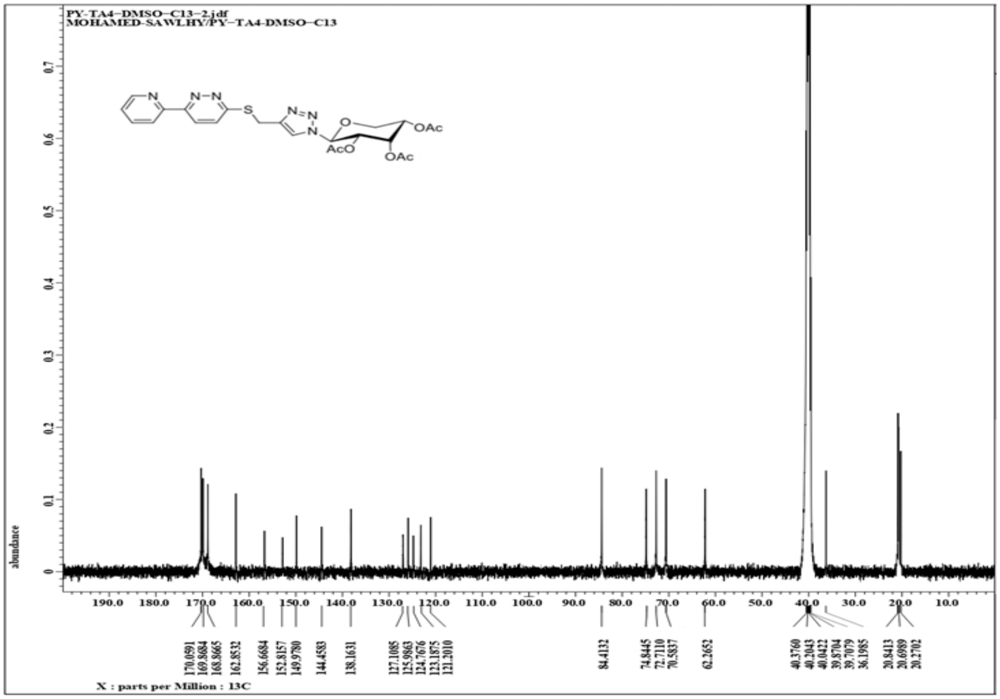

Figure S10: ^{13}C-NMR of Compound **6**

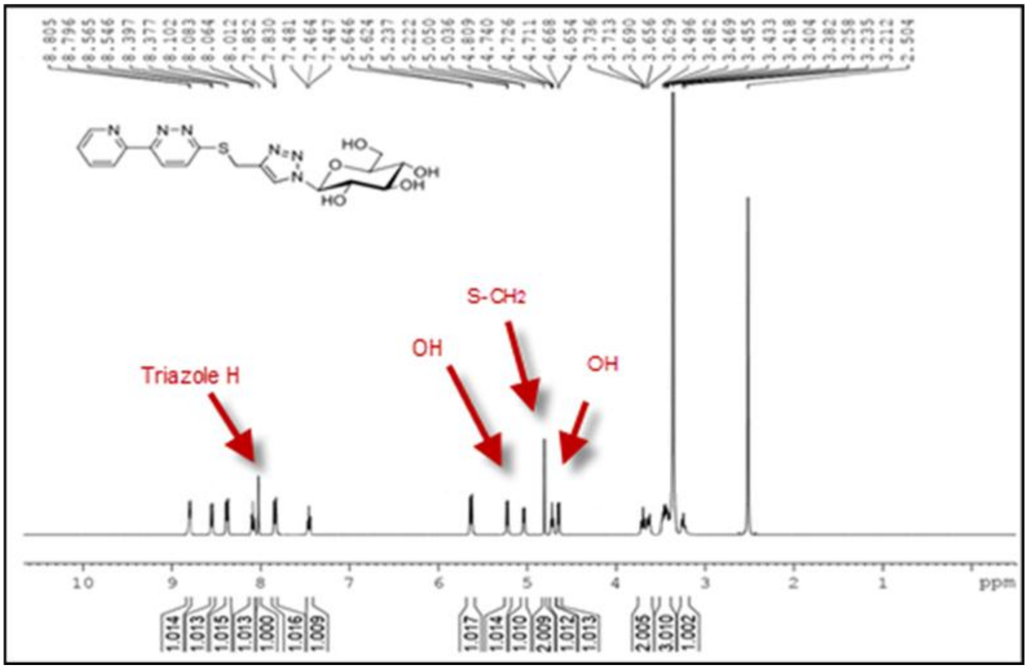

Figure S11: ^{1}H-NMR of Compound **7**

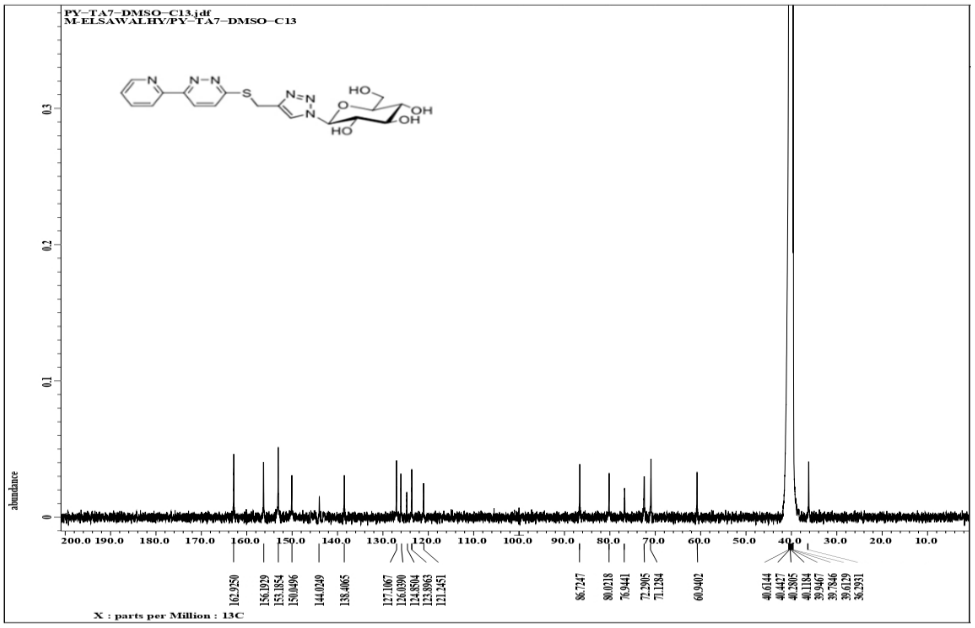

Figure S12: ^{13}C-NMR of Compound **7**

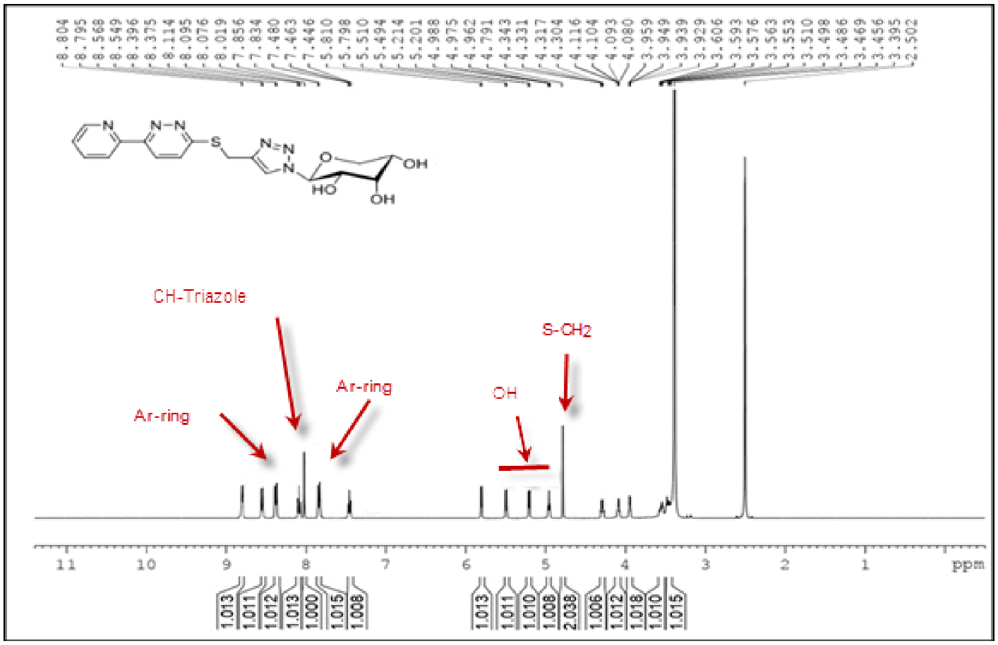

Figure S13: ^{1}H-NMR of Compound **8**

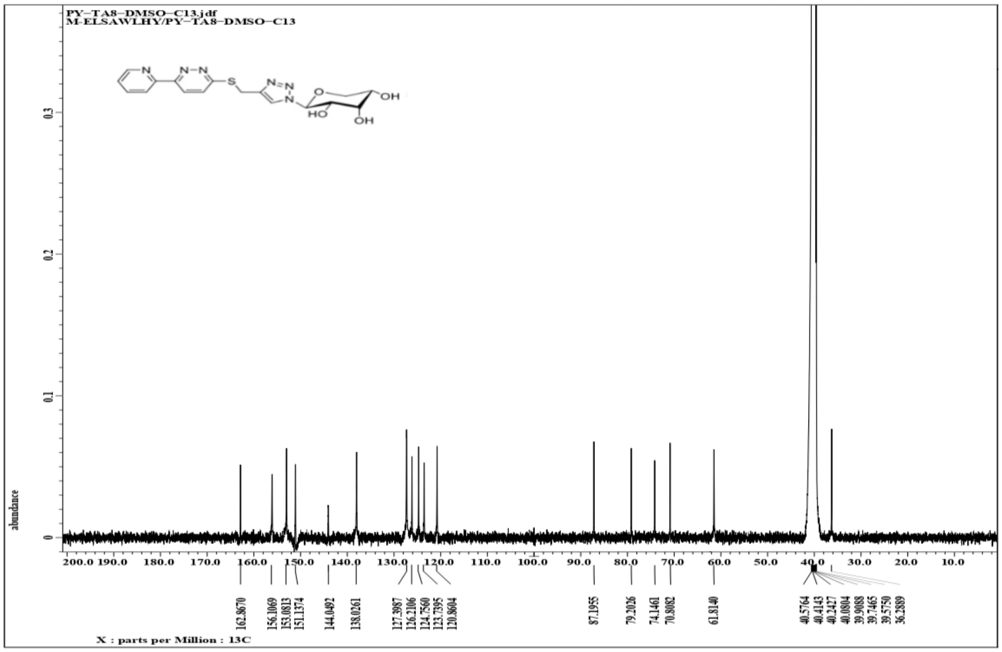

Figure S14: ^{13}C-NMR of Compound **8**

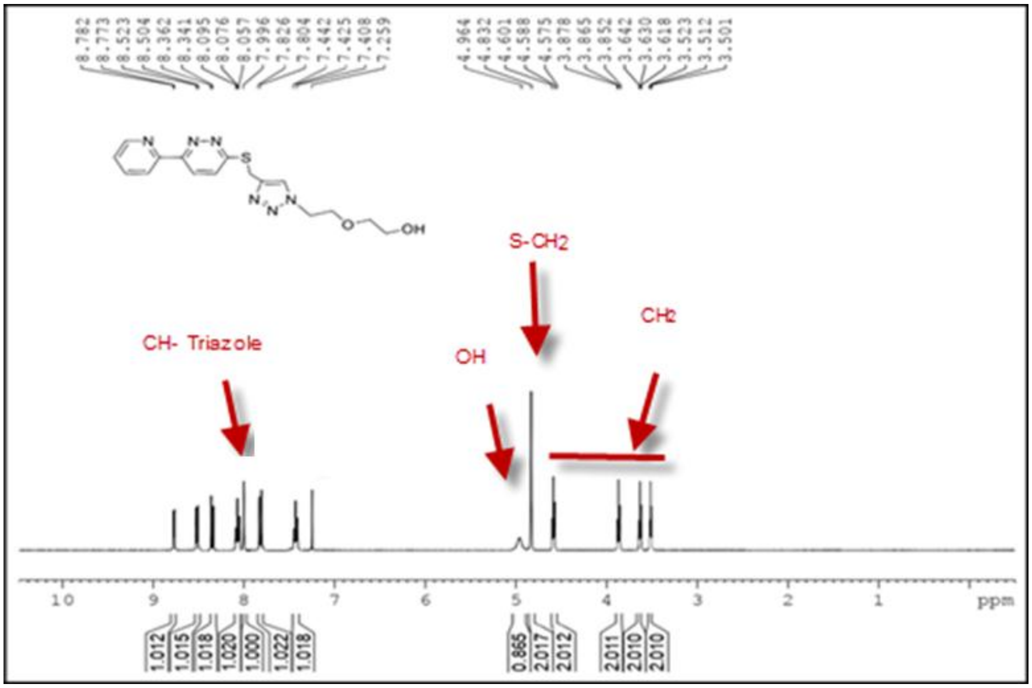

Figure S15: ^{1}H-NMR of Compound **9**

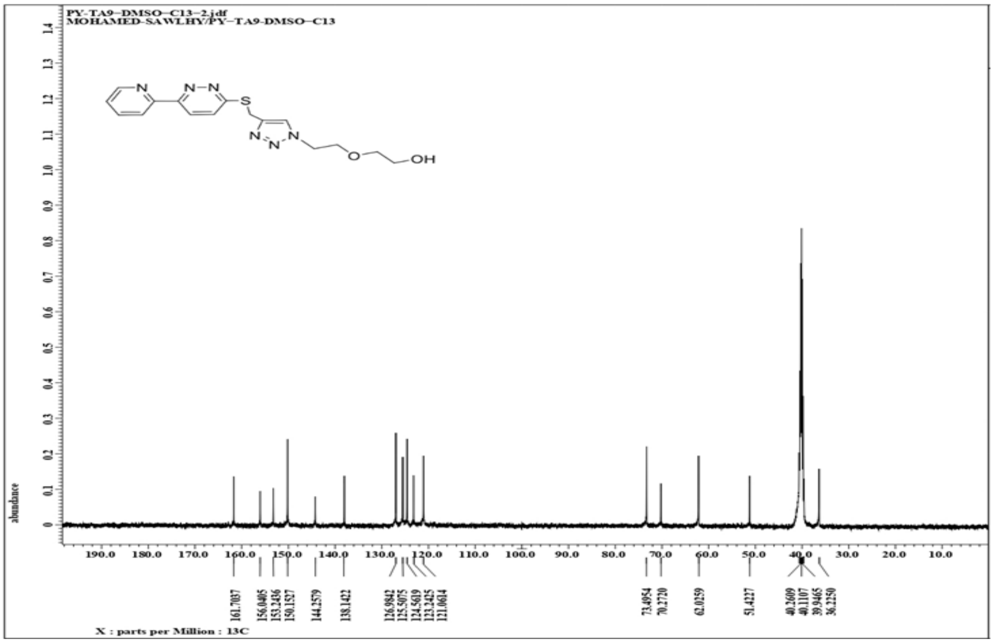

Figure S16: ^{13}C-NMR of Compound **9**

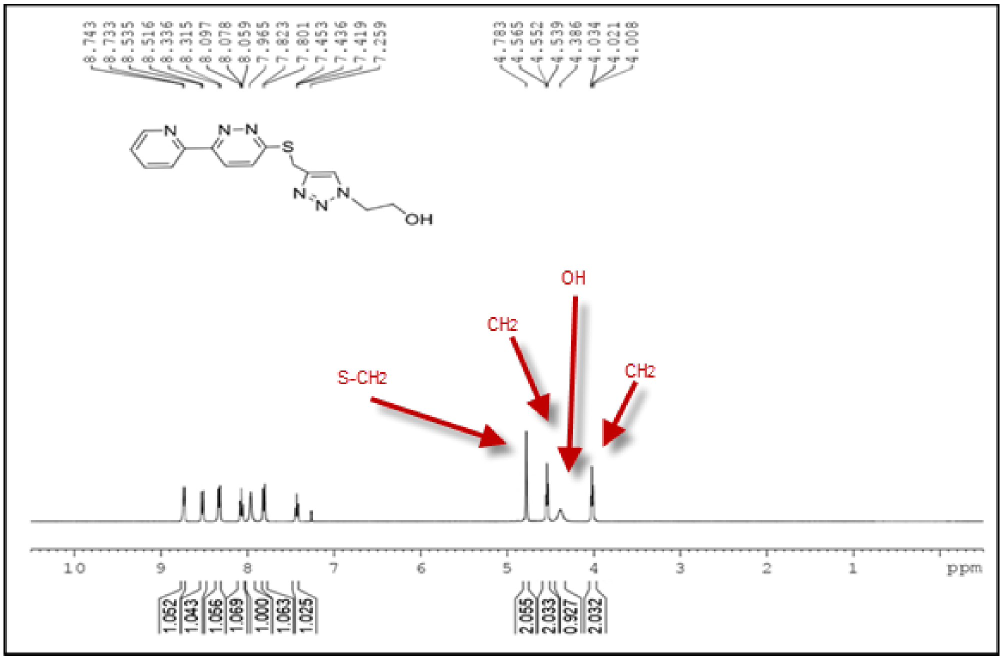

Figure S17: ^{1}H-NMR of Compound **10**

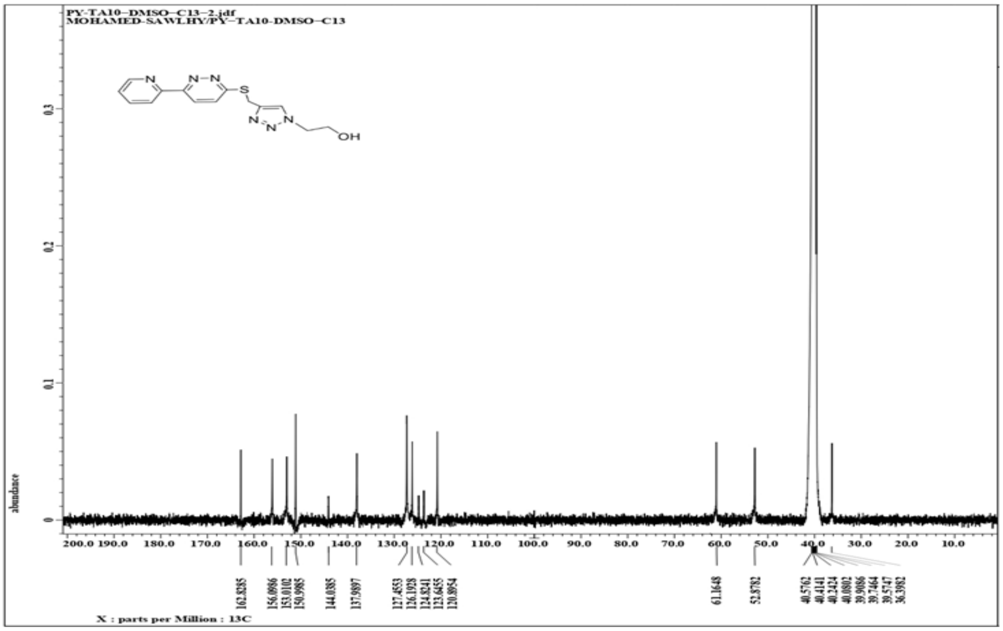

Figure S18: ^{13}C-NMR of Compound **10**

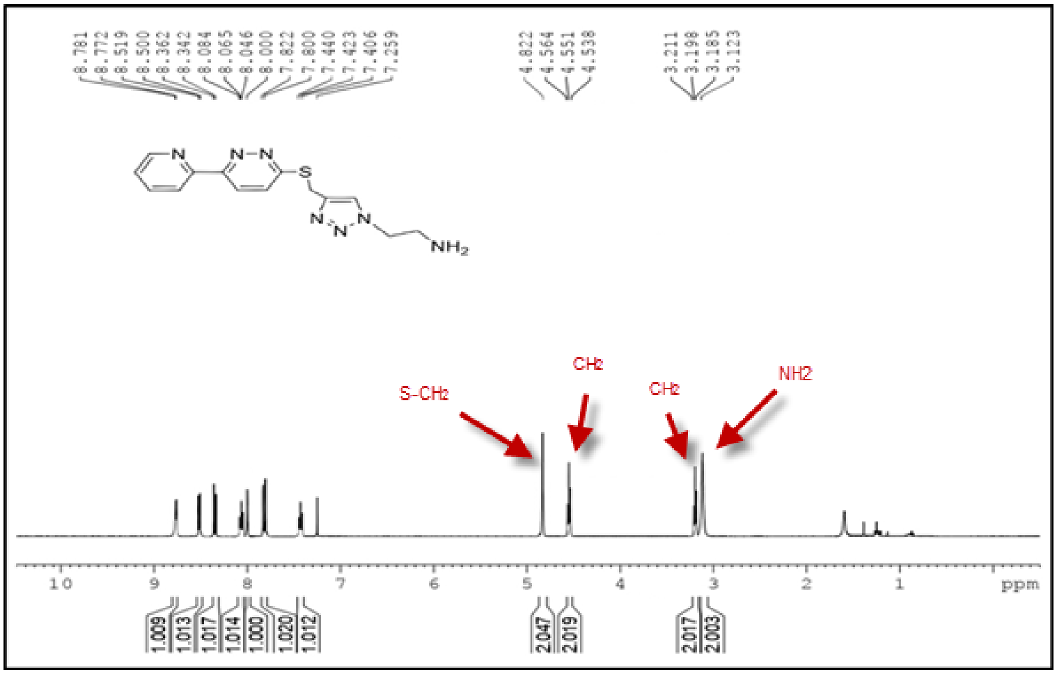

Figure S19: ^{1}H-NMR of Compound **11**

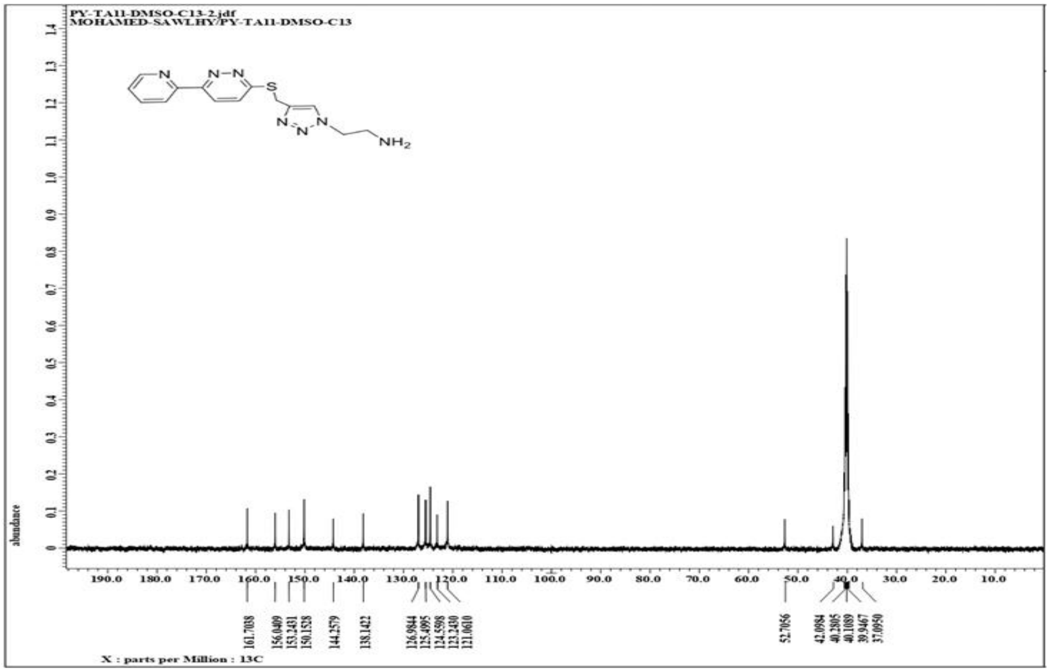

Figure S20: ^{13}C-NMR of Compound **11**

Table S1. AChE and BuChE inhibitory activities of synthesized compounds at 0.01, 0.1, 1.0, 10 and 100 μM.

Compounds	% inhibition 0.01 μM AChE(BuChE)	% inhibition 0.1 μM AChE(BuChE)	% inhibition 1 μM AChE(BuChE)	% inhibition 10 μM AChE(BuChE)	% inhibition 100 μM AChE(BuChE)
**1**	6.1(8.7)	23(19)	51(27)	74(54)	89(83)
**3**	7.4(25)	23(39)	43(61)	72(79)	88(93)
**4**	11(5.1)	29(11)	47(20)	71(41)	87(75)
**5**	42(25)	61(48)	71(67)	83(78)	94(91)
**6**	9(4.0)	29(8.1)	55(19)	73(33)	88(69)
**7**	14(7.8)	38(13)	62(23)	80(48)	92(79)
**8**	26(5.9)	43(13)	62(22)	88(55)	93(82)
**9**	35(22)	54(37)	70(56)	82(80)	92(93)
**10**	24(5.6)	40(13)	68(31)	83(51)	90(78)
**11**	7.5(−8.1)	26(7.5)	53(12)	76(33)	90(71)
**Donepezil**	45(16)	60(32)	72(65)	89(84)	95(94)
**Tacrine**	17(30)	35(48)	59(67)	81(84)	93(94)
**Rivastigmine**	6.9(0.7)	16(3.2)	30(7.0)	64(38)	87(80)
